# Experimental methods modestly impact interpretation of the effect of environmental exposures on the larval zebrafish gut microbiome

**DOI:** 10.1038/s41598-022-18532-x

**Published:** 2022-08-25

**Authors:** Keaton Stagaman, Kristin D. Kasschau, Robyn L. Tanguay, Thomas J. Sharpton

**Affiliations:** 1 Department of Microbiology, Oregon State University, Corvallis, OR USA; 2 Sinnhuber Aquatic Research Laboratory, Department of Environmental Toxicology, Oregon State University, Corvallis, OR USA; 3 Department of Microbiology & Department of Statistics, Oregon State University, Corvallis, OR USA

**Keywords:** Microbiome, Zebrafish

## Abstract

Rapidly growing fields, such as microbiome science, often lack standardization of procedures across research groups. This is especially the case for microbiome investigations in the zebrafish (*Danio rerio*) model system, which is quickly becoming a workhorse system for understanding the exposure-microbiome-physiology axis. To guide future investigations using this model system, we defined how various experimental decisions affect the outcomes of studies on the effects of exogenous exposure on the zebrafish gut microbiome. Using a model toxicant, benzo[a]pyrene (B*a*P), we assessed how each of two dissection methods (gut dissection vs. whole fish), three DNA extraction kits (Qiagen Blood & Tissue, Macherey–Nagel NucleoSpin, and Qiagen PowerSoil), and inclusion of PCR replicates (single vs. pooled triplicate reactions) affected our interpretation of how exposure influences the diversity and composition of the gut microbiome, as well as our ability to identify microbiome biomarkers of exposure. We found that inclusion of PCR replicates had the smallest effect on our final interpretations, and the effects of dissection method and DNA extraction kit had significant effects in specific contexts, primarily in the cases of identifying microbial biomarkers.

## Introduction

The zebrafish (*Danio rerio*) has become an increasingly popular model organism for the study of the gut microbiome, especially in the context of exposure science^1^. Zebrafish are among the most widely used model systems in biomedical research due to the high numbers of individuals that can be produced, the low cost of maintenance, their rapid maturation, and the extensive molecular tools that are available to the system (e.g., CRISPR/Cas9 gene editing). Moreover, zebrafish embryos can be isolated and experimentally manipulated in vitro, affording opportunities to discern how exposure to various exogenous factors, such as environmental toxicants, impacts zebrafish development. Accordingly, zebrafish have become a workhorse model for screening for the impacts of chemical and drug exposure on vertebrate physiology, behavior, and development. Recent years have witnessed a rapid integration of microbiome research methods into this model system, including relatively straightforward procedures for deriving germ-free and gnotobiotic zebrafish^2^, techniques for visualizing gut microbiota in situ^3^, and procedures for passive sampling of zebrafish microbiota^4^. The zebrafish model system has been especially useful for determining how chemical exposure impacts initial microbiome assembly^5^, alters established microbiota^6^, and how variation in the microbiome links to vertebrate physiology^7^. Given the utility of this model system and the advent of these microbiome approaches, the zebrafish affords researchers tremendous opportunity to disentangle the interaction between exogenous factors, the gut microbiome, and vertebrate physiology.

But despite this rapid growth—and possibly in part because of it—there has not been a consistent set of methods used across studies for assessing the composition and diversity of the zebrafish gut microbiome. This can be due to a number of factors including differences in budget, cultural inertia, and simply the heterogenous adoption of innovations that arise with some frequency in such areas of growth. In particular, researchers will extract DNA either from whole fish carcasses (which likely include microbes residing in non-intestinal tissues) or extracted intestinal tissue, use different DNA extraction kits, or implement measures to correct for PCR bias (e.g., single vs. pooled triplicate PCR replication). Prior work in other research systems has suggested that experimental decisions can impact discovery in microbiome investigations^8–14^, and different experimental strategies may complicate data integration and interpretation across studies^15^. For example, an extensive array of studies have evaluated how the specific 16S hypervariable that is targeted during PCR as well as the specific primers that are used to conduct PCR impact study outcomes^16–21^. Moreover, some experimental decisions (e.g., triplicate PCR) can be expensive or time consuming, to the point that they work against the scalability that is inherent to the zebrafish research system, and may actually be unnecessary for some studies^22^.

To shed light on the impact of common experimental approaches in zebrafish gut microbiome research, we exposed fish to an environmental toxicant and determined how different experimental decisions impacted subsequent microbiome analyses. Specifically, we exposed 384 zebrafish embryos to either 10 µM benzo[*a*]pyrene (B*a*P) or an embryo medium (water plus salts) control and assessed how dissection method, DNA extraction kit, and PCR replication affected assessment of how B*a*P exposure impacts larval zebrafish gut microbiome diversity and composition.

## Methods

### Experimental methods

**Figure 1:**
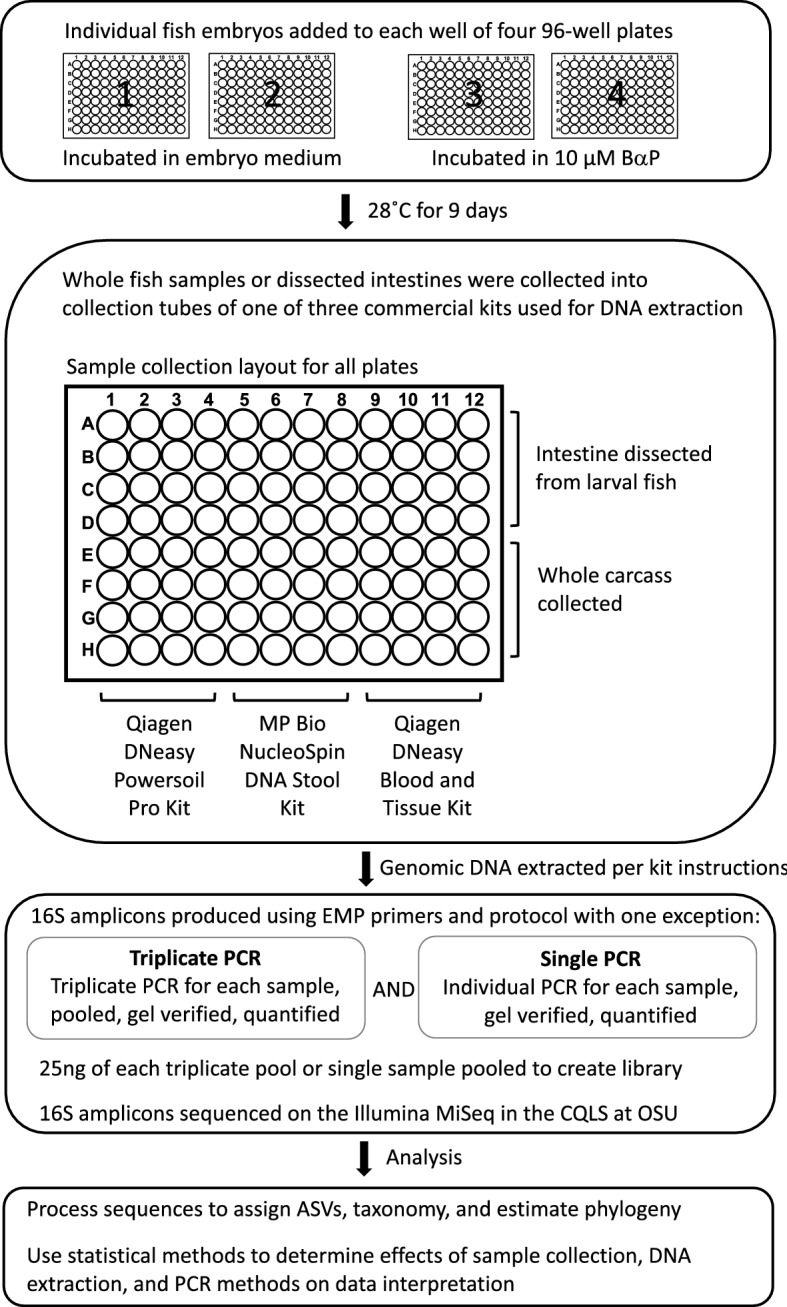
Experimental design. Overview of the methods analyzed for impact on interpretation of experimental results. First panel, 384 zebrafish larvae were distributed across the wells of four 96-well round-bottom plates. For two of these plates, the larvae were left untreated, while the larvae of the other two plates were exposed to 10 µM benzo-[a]-pyrene by way of their growth media. The zebrafish were raised in their wells on these plates until 9 days post fertilization. Second panel, for each of the four plates we conducted a fully factorial splitting of the samples by dissection method and DNA extraction kit. Half of the samples on each plate had their intestines dissected and placed in the collection tube, while for the other half, we placed the whole carcasses in their collection tubes. Then, in a manner orthogonal to dissection method, a third of each set of samples underwent DNA extraction using one of the three extraction kits. Third panel, we prepared 16S rDNA sequencing libraries using either single or triplicate PCR. Every sample was subject to both of these treatments. These libraries were then submitted for sequencing. Bottom panel, upon receiving the 16S sequences, we processed the data for quality and conducted the analyses described herein.

Figure 1 graphically details the methods workflow. Zebrafish embryos were provided by the fish laboratory at the Sinnhuber Aquatic Research Laboratory at Oregon State University. All experimental protocols and methods were carried out in accordance with the animal use and care protocol (# 5068) approved by the Institutional Animal Care and Use Committee at Oregon State University, and in accordance with the ARRIVE guidelines. Prior to this experiment, we conducted a pilot power analysis to determine how many samples we would need to reliably detect the effects of 10 µM B*a*P exposure on the larval zebrafish intestinal microbiome in terms of both alpha- and beta-diversity. Results from that power analysis indicated we would need to include four 96-well plates worth of fish (significance threshold set at 0.05, statistical power set at 0.8).

Tropical 5D strain zebrafish were reared in Sinnhuber Aquatic Research Laboratory under standard laboratory conditions of 28 °C on a 14 h light/10 h dark photoperiod according to standard zebrafish breeding protocols^23^. All fish embryos were collected on a single day and added to each well of four 96-well plates. Embryos were maintained in their wells for the duration of the experiment for 9 days. Two plates of embryos were exposed to 10 µM B*a*P while the other two plates were only exposed to the embryo medium. We chose 10 µM as the dose for BaP exposure because our prior work that explored the effect of different concentrations of BaP exposure on zebrafish behavior found that 10 uM embryonic exposure elicits known effects on behavior in zebrafish while avoiding other major toxicity effects^24^. Furthermore, from our pilot power analysis, we also determined that detection of microbial disruption due to B*a*P exposure was most reliable at this concentration compared to 1 and 5 µM concentrations. At 9 days post fertilization (dpf), fish were euthanized by placing the 96-well plates in an ice slurry (the wells of the plates were submerged, but whole plates themselves were not, to prevent non-sterile water from entering the wells).The intestines were dissected from half the larval fish on each plate of untreated and B*aP* treated larval fish; these intestines were used for microbiome sample processing for these individuals. For the remaining untreated and B*a*P treated embryos, whole carcasses were used for microbiome sample processing. Dissected intestines and whole fish were placed directly into extraction tubes containing DNA-stabilizing buffer. Once a batch of four or five samples were in the extraction tubes, that batch would be flash frozen in liquid nitrogen, so that no sample was in the extraction tube but unfrozen for more than about five minutes. We did this was for two reasons: (1) the flash freezing can help with physical lysing of the bacterial cells for better DNA extraction, and (2) the facility in which we collected samples was not the same facility in which we were going to conduct the extraction and PCR (these two facilities are roughly 4 miles apart. So, we kept the sample frozen and stabilized in the buffers to prevent alterations in the microbiome post sampling.

Randomizing across plates and dissection methods, three different, commonly utilized commercial kits were used to extract DNA, these included Qiagen DNA Powersoil Pro Kit (Cat. No. 47016), Qiagen DNA Blood & Tissue Kit (Cat. No. 69506) and Macherey–Nagel NucleoSpin DNA Stool (Cat. No. 740472). We selected these kits because they represent either a relatively standard and widely employed approach, as in the case of the Powersoil Pro kit, which kit used by the Human Microbiome Project^25^ and Earth Microbiome Project^26^, or because they offer opportunities to improve the scale of an investigation due to decreased costs (e.g., Blood & Tissue and NucleoSpin). In particular, the Blood & Tissue kit is produced by the same manufacturer as the Powersoil kit, but does not include bead beating and is 50% of the cost, which are features that could improve experimental throughput and scale. The Nucleospin kit, like the Powersoil kit, uses beads to mechanically disrupt cells, but is a significantly less expensive alternative.

Dissected intestines or whole carcasses were collected into the bead tube and extraction buffer provided by each kit for Qiagen DNA Powersoil Pro Kit and Macherey–Nagel NucleoSpin DNA Stools kits. Samples were collected into 1.5 ml microfuge tubes with lysis buffer for the Qiagen DNA Blood & Tissue Kit. All samples were frozen in Liquid Nitrogen after tissue collection and stored at -80˚C until extractions were performed. DNA extracts of each sample were split into four aliquot, each of which were then subject to PCR amplification of the V4 region of the 16S rRNA gene following our prior approaches. We also include negative control samples including only buffer or water from the DNA extraction kits and the PCR reagents in the sequencing libraries. Briefly, using the Earth Microbiome Project (EMP) 16S V4 amplification index primers 515F and 806R, DNA was amplified following a slightly modified EMP PCR reaction mixture protocol. That is, We followed the Earth Microbiome Project (EMP) PCR protocol^26^ to amplify the V4 subregion of the 16S rRNA gene, but made slight modifications to the protocol to facilitate accurate pipetting. These modifications did not change the overall chemistry of the reactions as compared to the EMP approach. The 515F barcoded primer was diluted to 2.5 µM and 2.0 µl of this solution was added to each PCR reaction. The 806R primer was diluted to 10 µM and 0.5 µL of this solution was added to each PCR reaction. 1.0 µl of extracted DNA was added to each PCR reaction. PCR was run on the Thermofisher SimpliAmp Thermocycler with the following conditions: 94 °C, 3 min; 35 cycles of 94 °C, 45 s; 50 °C 60 s; 72 °C 90 s; final 72 °C, 10 min. Of the four aliquots for each sample, three were pooled (triplicate reaction), as is commonly done in microbiome research to account for possible PCR bias, and the fourth was directly submitted for sequencing.

All PCR products were run on 1.5% agarose gel in 0.5X TBE running buffer to verify PCR product size and negative controls. PCR products were size selected using a BluePippen at the Oregon State University Center for Quantitative Life Sciences. The PCR products in the 300-600nt range were selected to remove a 110-120nt host contaminate PCR product. PCR products were quantified using the Qubit dsDNA HS Assay Kit (Thermofisher Q32854) and read on a Qubit 2.0. Equal nanograms of PCR product for each sample were combined to make a library pool, the pools were processed through the QIAquick PCR Purification Kit (Qiagen ID # 28,104), and final quality control checked on the Agilent Tapestation 4200 in the CQLS. PCR libraries were submitted to the Oregon State University Center for Quantitative Life Sciences and sequenced on their Illumina MiSeq System (RRID:SCR_016379) using v3 chemistry to produce 300 bp paired-end reads.

### Sequence processing

We used the dada2^27^ *R*^28^ package to quality filter, merge reads, and assign amplicon sequence variants (ASVs) from the raw sequences, and to create an ASVs counts per sample table. None of the negative control samples from the extraction kits and PCR reagents passed the dada2 quality controls. We used dada2 in conjunction with the Silva 16S database^29^ to assign taxonomy to the ASVs down to the genus level. We used the NAST algorithm in mothur^30^ and guide sequences from the Silva 16S database to align ASV sequences and used FastTree^31^ to create an approximately-maximum likelihood phylogeny of the ASVs. ASVs that received no assignment at the kingdom taxonomic level or that were assigned Chloroplast or Mitochondria at the order or family taxonomic levels were removed from the analysis.

### Statistical methods

#### Alpha-diversity

We used the phyloseq^32^ and picante^33^ R packages to calculate alpha diversity metrics for each sample, which included Chao1 estimated abundance^34^, the Shannon diversity index^35^, the Simpson diversity index^36^, observed number of ASVs, and Faith’s phylogenetic diversity^37^. Because alpha-diversity scores are often not normally distributed, we used the functions descdist and fitdist (fitdistrplus package^38^) to determine that the best distribution for each alpha-diversity metric scores was almost always the beta distribution. This distribution is not directly supported by the glm function but is approximated by the quasibinomial family, which we used for all linear models unless otherwise noted. These distributions only take values from 0 to 1, so we divided all alpha-diversity scores by the max score for each metric. All samples underwent both single and triplicate PCR (though we did not necessarily get a set of sequences that passed QC and rarefaction for every set of single or triplicate PCR sample for every single biological sample), we first assessed whether there was a statistical interaction between B*a*P treatment and PCR method using a generalized linear mixed effects model (glmer.nb; lme4 package^39^) with the zebrafish sample ID as a random factor (e.g., Chao1_score ~ BaP_treatment * PCR_method + (1 | SampleID)). Because these models showed no significant effects of PCR method on our assessment of how B*a*P exposure affects alpha-diversity and in order not to artificially inflate our sample size with redundant samples, we randomly chose a single- or triplicate PCR sample as a representative for each zebrafish microbiome sample for the remaining analyses (i.e., determining whether extraction kit or dissection method affects the assessment of the effects of B*a*P on alpha-diversity), Furthermore, because random fish death and removal of samples due to poor sequencing quality was not exactly equally distributed, we subsampled an equal number of samples for each kit by dissection method by B*a*P exposure treatment so direct comparisons could be made between treatments without concerns over differences in sample numbers. For each biological sample, the R function sample was used to randomly choose the triplicate or single PCR reaction sample associated with it. For sub-sampling to even numbers between treatments, we took counts of each subset of interest (e.g., dissected-unexposed, dissected-exposed, whole-fish-unexposed, and whole-fish-exposed) and choose the subsampling N to be equal to the smallest of those subsets. Samples were then randomly chosen from each subset by the sample function. A breakdown of sample numbers by treatments can be found in (Supplemental Table 1).

### Beta-diversity

To assess differences in microbiome composition between samples (beta-diversity), we generated distance matrices for all samples using six different distance metrics: Bray-Curtis^40^, Canberra^41^, Sørensen^42^, weighted UniFrac^43^, half-weighted UniFrac^44^, and unweighted UniFrac^44^ (function gen.dist.matrices; phyloseqCompanion package^45^). Using these distance matrices, we built full dbRDA models (function capscale from the vegan package^46^) and then assessed significance with anova.cca (a PERMANOVA model; vegan package). We used the function get.biplot.data from the phyloseqCompanion and ggplot (ggplot2 package^47^) to create ordinations based off of the various distance metrics we used. As with alpha-diversity, we first tested the effect of PCR method using a PERMANOVA model wherein we used zebrafish sample ID as a blocking factor (permutations only occurred within each ID rather than across all samples), a non-parametric analog for a random factor in a parametric test. We then proceeded with the same sub-sampled sets from the alpha-diversity analyses to test whether extraction kit or dissection method affects the assessment of B*a*P exposure effects on beta-diversity.

### Taxonomic indicator analyses

We used LEfSe (Linear discriminant analysis Effect Size)^48^ as implemented in Galaxy (Huttenhower lab; http://huttenhower.sph.harvard.edu/galaxy/) to determine which taxa were statistically significant indicators of B*a*P exposure in our various subsets. We used LEfSe to identify taxa that indicated B*a*P exposure in the dissected intestinal samples and repeated this analysis using only the samples obtained from whole fish. Similarly, samples from each set of DNA extraction procedures were used to identify taxa that indicate B*a*P. These sets of indicator taxa were then compared across the LefSe tests to resolve taxa that indicate exposure irrespective of dissection approach or DNA extraction procedure. All analyses were run with the default parameters and with B*a*P treatment as class and sample ID as subject.

### Random forest analyses

As with LEfSe, we generated random forest models for each experimental subset of samples (i.e., dissection methods and extraction kits) using the caret package^49^ for training the model and the ranger package^50^ for implementation of the random forest method. Prior to running the random forest model, we removed taxa with near-zero variation using the step_nzv function (recipes package^51^). We used fivefold cross validation and the ROC metric for choosing the best model. The function roc (pROC package^52^) was used for calculating the ROC curves of the final models and these data were used for plotting with ggplot. Importance values for all taxa in the random forest models were calculated during model training and significance of the importance values was determined using the permutation approach^53^.

### Logistic regressions

Taxa deemed significantly important in each random forest model were then used to build logistic regression models to predict B*a*P exposure from their relative abundances. Models were built in forward step-wise manners, starting with the most important taxa from each model. Models were built with the glm function and each new model was compared to the previous with anova (both functions from the base stats package). New models (i.e. models with an additional term) that were statistically better than the previous model were kept as the current model and models that were not significantly better were discarded. Taxa that were assessed as significantly predicting B*a*P exposure in the final model (Anova from the car package^54^ with type II tests) were considered indicators.

## Results

In order to test our hypotheses, we raised zebrafish for 9 days, with one fish in each well of four 96-well plates in 100 µL of embryo medium. The embryos in two of the plates also received exposure to benzo-[a]-pyrene at a concentration of 10 µM. We divided the experimental methods the samples underwent in a fully factorial manner for dissection method and extraction kit, and every sample underwent both single and triplicate PCR. We began our analysis by assessing the impact of PCR approach—namely how the number of replicate PCR—impacts the relationship between microbiome alpha-diversity and exposure to B*a*P. Based on linear regression, we observed no statistically significant effects of single versus triplicate PCR on the relationship between B*a*P treatment and any metric of alpha-diversity (Supplemental Fig. 1 and Supplemental Table 2). Moreover, samples treated with B*a*P exhibited consistently higher alpha-diversity scores regardless of PCR method (Supplemental Fig. 1). These results indicate that single PCRs reveal consistent associations between alpha-diversity and exposure as those obtained from triplicate PCRs.

**Figure 2:**
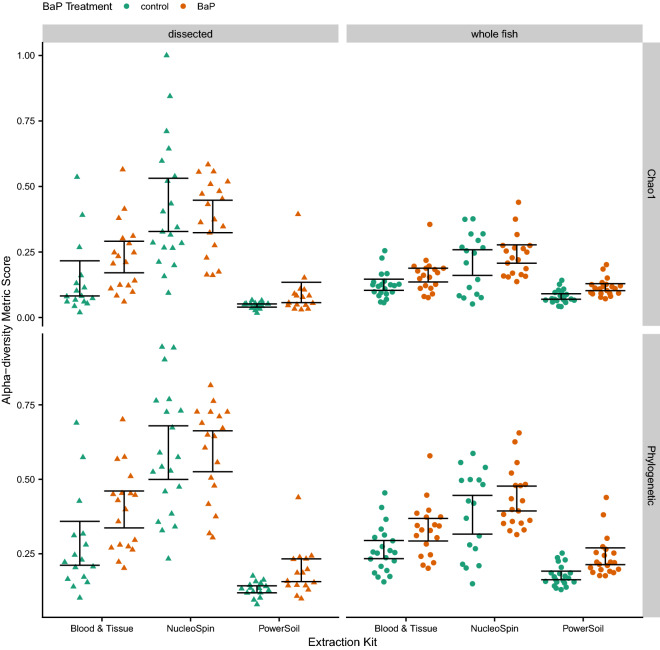
Swarm plots of Chao1 and Phylogenetic alpha-diversity metrics by dissection method and DNA extraction kit. The color of the point indicates BaP treatment (unexposed control vs. exposed). The black error bars indicate bootstrapped 95% C.I.s of the means.

For our other assessments of methodology effects (i.e., dissection and DNA extraction approaches), we needed to make sure we were not artificially increasing our sample size by including both single and triplicate PCR reactions in our analyses, since nearly all samples went through both methods of PCR. So, we randomly chose a single or triplicate PCR reaction sample to represent each biological zebrafish microbiome sample in the subsequent analyses. We also randomly selected a consistent number of samples across each group subject to comparison to avoid biases due to inconsistent sample sizes. Unlike the PCR results, these analyses reveal significant main effects of B*a*P treatment, DNA extraction kit, and dissection method across all of the diversity metrics we assessed. The Chao1 and richness metrics were also associated with the interaction between extraction kit and B*a*P treatment (Supplemental Table 3). These results imply that, while extraction kits and dissection methods influence the measurement of microbiome diversity, they do not generally do so in a manner that would alter our interpretation of whether B*a*P treatment affects microbiome diversity (i.e., the direction of the effect). As noted with the PCR methods analysis, B*a*P treatment consistently increases alpha-diversity regardless of methods used (Fig. 2).

**Figure 3:**
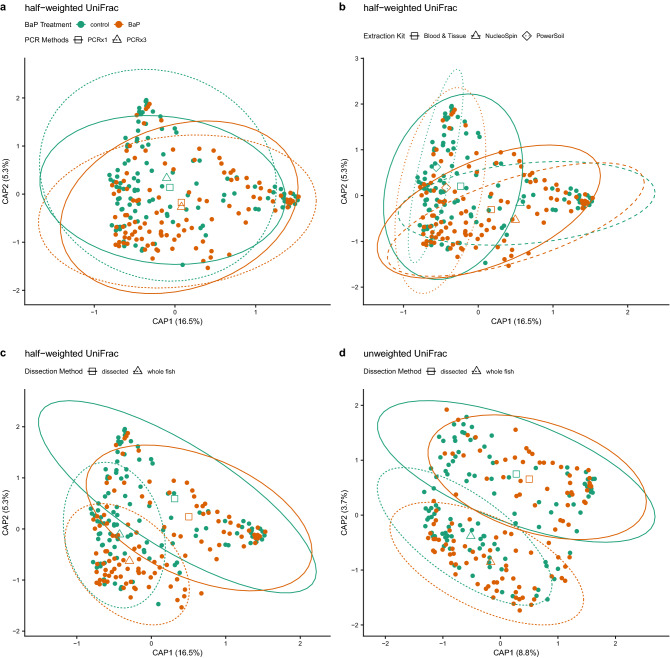
dbRDA ordinations of differences in zebrafish microbiome composition (beta-diversity) highlighting various significant associations between differences in composition and methodology. In all panels, solid points are colored by BaP treatment, open shapes (triangles, diamonds, squares) indicate centroids of each BaP treatment by processing method interaction, and ellipses demarcate 95% C.I.s for these centroids. Panels a-c depict identical half-weighted UniFrac ordinations with different statistical associations highlighted. (**a**) BaP exposure and PCR replication (1x vs. 3x); open squares and solid lines indicate centroids and their 95% C.I.s for single PCR samples, open triangles and dashed lines indicate centroids and their 95% C.I.s for triplicate PCR samples. (**b**) BaP exposure and DNA extraction kit; open squares and solid lines indicate centroids and their 95% C.I.s for Blood & Tissue-extracted samples, open triangles and dashed lines indicate centroids and their 95% C.I.s for NucleoSpin-extracted samples, and open diamonds and dotted lines indicate centroids and their 95% C.I.s for PowerSoil-extracted samples. (**c**) BaP exposure and dissection method; open squares and solid lines indicate centroids and their 95% C.I.s for dissected intestine samples, open triangles and dashed lines indicate centroids and their 95% C.I.s for whole fish samples. Panel (**d**) depicts an unweighted UniFrac ordination with the statistical interaction between BaP and dissection method highlighted with the same information overlaid as in panel (**c**).

We next sought to determine if the assessment of how B*a*P exposure impacts microbiome composition is influenced by sample preparation methodology. To do so, we conducted statistical assessments of microbiome beta-diversity and did so using a variety of diversity metrics. Unlike with alpha-diversity, the beta-diversity analyses do reveal a statistically significant main effect of single versus triplicate PCR on assessing microbiome composition, indicating that the inferred microbiome composition from an analysis is influenced to some extent by whether one or three PCRs were applied to a sample. However, except for the Sørensen metric (a taxonomic presence/absence metric), there are no significant interactions between B*a*P treatment and PCR method predicting microbiome composition (Supplemental Table 4). Moreover, the model coefficient for the effect of the number of PCRs is far smaller than the coefficient for the effect of B*a*P treatment. Additionally, distance-based redundancy analysis (dbRDA) ordinations of beta-diversity reveal very similar centroids for B*a*P treatments, regardless of PCR method (Fig. 3a). Collectively, these results indicate that the number of PCRs applied to a sample elicit a small, technical effect on the results that does not obscure or interfere with the effect due to toxicant exposure, at least in the case of B*a*P.

For all six beta-diversity metrics assessed, there are significant main effects of B*a*P treatment, extraction kit, and dissection method. There are, however, no statistically significant interactions between B*a*P treatment and either extraction kit or dissection method for four of the six beta-diversity metrics; the Canberrra and unweighted UniFrac metrics are associated with the interaction between B*a*P treatment and dissection method (Supplemental Table 5). Collectively, these results indicate that extraction kit and dissection method do have an effect on the inference of the zebrafish gut microbiome composition (Fig. 3b–d). Notably, the dissection method and extraction kit elicit a larger effect than B*a*P exposure on the composition of the gut microbiome, as determined by PERMANOVA model coefficients. However, whether these experimental parameters impact a given study’s ability to determine whether B*a*P exposure affects the gut microbiome depends upon the specific beta-diversity metrics being used. In particular, metrics that emphasize the contribution of rarer members of the microbiome may be more sensitive to these experimental parameters than those that emphasize the contribution of the more abundant microbiota in the community.

**Figure 4:**
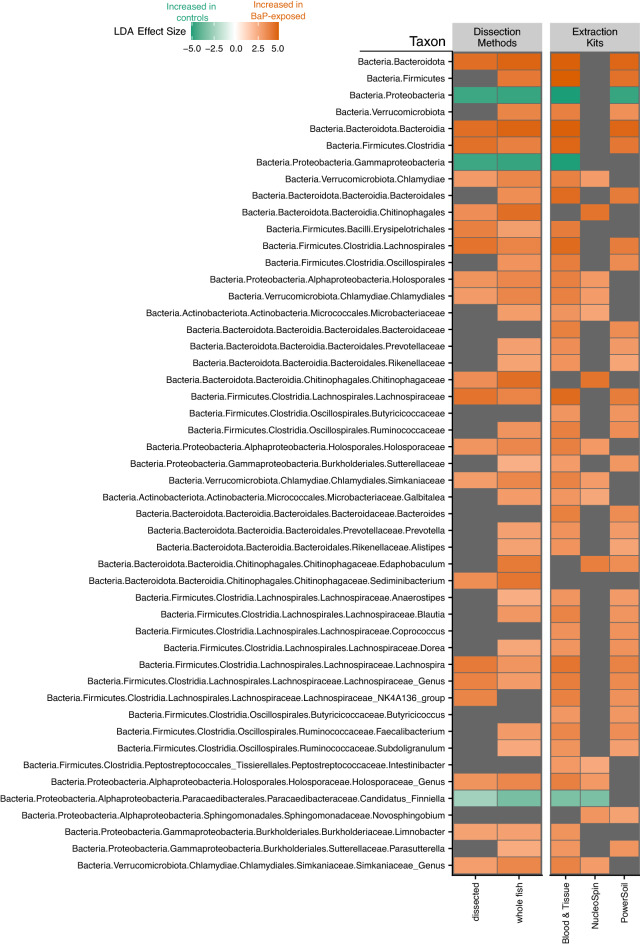
Heatmap of statistically significant effect sizes estimated by LEfSe. This plot only shows phylotypes that were consistently identified as indicators across at least two data sets. The color of the square indicates the effect size with green meaning a phylotype is indicative of the control (no BaP exposure) while orange means a phylotype is indicative of BaP exposure. Gray squares indicate the taxon is not an indicator for either the control or treatment for that data set.

In addition to overall microbiome diversity and composition, we aimed to determine how different methods impact which taxa were found to have significant associations with B*a*P treatment. We used LEfSe, a hierarchical linear discriminant analysis, to determine which phylotypes are significant indicators of B*a*P exposure using different data sets, such as taxa that differentiate B*a*P exposure in dissected versus whole fish samples, or those taxa that differentiate B*a*P exposure in samples associated with specific DNA extraction kits. A few phylotypes are consistently indicative of no exposure—e.g., Protobacteria, Gammaproteobacteria, Paracaedibacteraceae—and several are indicative of B*a*P exposure—e.g., Bacteroidia*,* Clostridia, Chitinophagaceae—across at least two data sets (Fig. 4). However, most phylotypes that were identified as an indicator in one data set were not consistent indicators across other data sets (Supplemental Fig. 2). In order to assess whether there were any interactions between dissection method and extraction kit in terms of this indicator analysis, we ran additional LEfSe analyses looking at sample subsets divided by both dissection method and extraction (e.g., assessing dissected samples extracted with the Blood & Tissue kit only). Of note, only the Blood & Tissue kit extract samples returned significant indicators for both dissected and whole fish samples, while for NucleoSpin it was just the dissected samples and for PowerSoil it was just the whole fish samples (Supplemental Fig. 3). The lack of significance for two of the extraction kits may very well be a statistical artifact due to how low we had to subsample for each of these dissection method by extraction kit groups. All together, these observations suggest that the identification of specific microbial indicators of exposure is sensitive to experimental decisions, which holds implications for how data generated under different experimental conditions is integrated or compared.

**Figure 5:**
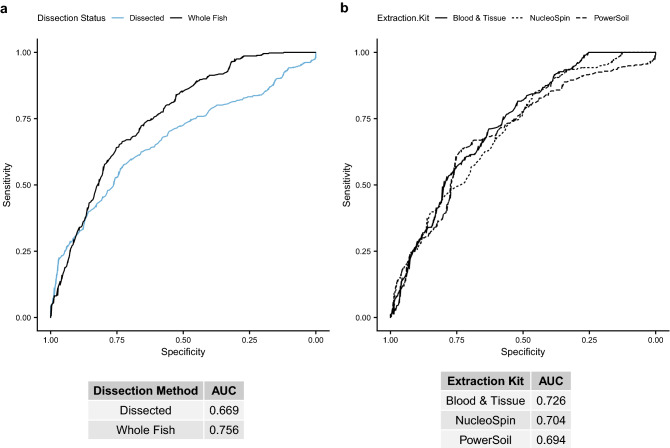
Receiver operating characteristic (ROC) curve plots for random forest models predicting BaP exposure from various data subsets, indicated by line color or line type, of centered log-ratio transformed ASV counts. Tables under each panel indicate the area under the ROC curve (AUC) for each model. A higher AUC indicates a better model, with an AUC of 0.5 indicating a 50% chance (essentially a random guess) of the model correctly predicting BaP exposure from taxon abundances. (**a**) ROC curves for dissected intestine samples (blue line) versus whole fish samples (black line), with the areas under the curve (AUCs) listed in the table below. (**b**) Similar data as in panel a, but for subsets by DNA extraction kit: solid line = Blood & Tissue, short dashed line = NucleoSpin, long dashed line = PowerSoil.

Groups of microbes, or consortia, may collectively indicate an exposure with higher sensitivity than specific microbial clades. So, we used random forest machine learning to identify consortia of ASVs that discriminate samples by their B*a*P exposure status using different subsets of samples, i.e., dissected versus whole fish samples, or the samples that underwent DNA extraction with each kit. The random forest models identified between 722 and 1578 ASVs, depending on the data set, that were deemed significantly important, and which we included in subsequent logistic regression analyses. We also used the models to produce ROC curves to see which data sets were better at predicting B*a*P treatment from ASV abundances. Model performance is assessed as the area under the curve (AUC) under the ROC curve. The whole fish data set had a greater AUC (0.756) than the dissected guts data set (0.669; Fig. 5a). For the extraction kits, the Blood & Tissue kit data set best discriminated controls from B*a*P treatment through this approach (AUC 0.726, other kits < 0.71; Fig. 5b). It should be noted that for these analyses, we used centerlog-ratio (CLR) transformed counts for the ASV abundances as we knew we would be continuing these analyses with regression models, and CLR transformation of abundance data can aid in preventing the identification of spurious associations^55^. However, we also built models using rarefied ASV counts that had greater AUCs for all data sets (Supplemental Fig. 4). Furthermore, it is important to note the distinction between accurately imputing the taxonomic composition of the community and resolving differences in the compositions between communities; it is possible that biases in an extraction procedure create the appearance of large differences between communities when in actuality the differences are relatively muted. Such phenomena could arise if kits fail to recover DNA from an important fraction of the microbial community due to extraction biases and if that fraction of the community is generally resistant to the variable being evaluated (e.g., B*a*P).

**Figure 6:**
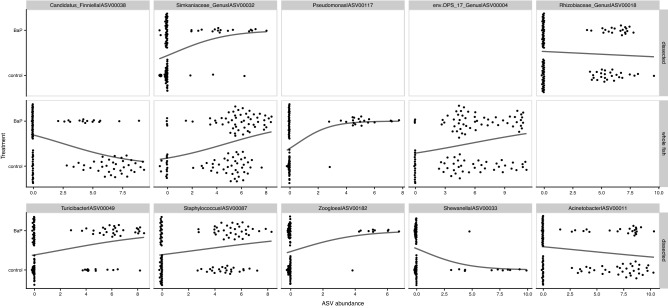
Swarm plots of logistic regressions predicting BaP exposure from individual ASV abundances (centered log-ratio transformed) using data subsets partitioned by dissection method. The top 10 taxa, ordered by importance according to the preceding random forest analysis are presented in this figure. Points indicate an ASV’s abundance per each dissection method. Black lines indicate the predicted relationship taken directly from the logistic regression models.

In order to determine how dissection method influences our interpretation of how individual microbial taxa associate with BaP exposure, we built binomial generalized linear models predicting BaP treatment from abundances of the ASVs deemed significantly important for either the dissection-only or whole fish random forest models (two total linear models; Supplemental Table 6). ASVs were evaluated if they improved the performance of a forward-built model, wherein one model parameter (i.e., ASV) is added to the model in series and compared to prior models through a Chi-square test to assess model optimality. The abundances of only one ASV, ASV00032, which is assigned to an uncharacterized genus in the Simkaniaceae family, significantly predicted B*a*P exposure in both data sets (its abundance is positively associated with B*a*P exposure; Fig. 6; Supplemental Fig. 6; Supplemental Table 7). The abundances of 27 taxa significantly predicted exposure in just one data set or the other (Supplemental Fig. 6). In short, the dissection approach used in the study impacts findings of which taxa in the microbiome link to exposure and, in some cases, how they do so.

**Figure 7:**
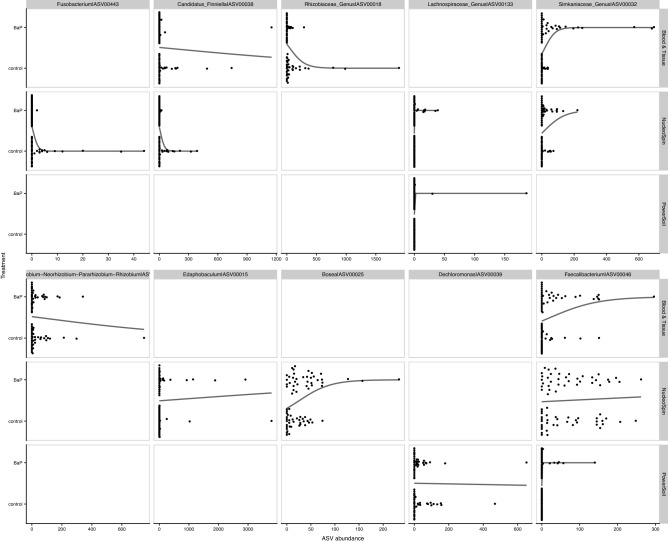
Swarm plots of logistic regressions predicting BaP exposure from individual ASV abundance (centered log-ratio transformed) using data subsets partitioned by dissection method. The top 10 taxa, ordered by importance from the preceding random forest analysis are presented in this figure. Points indicate an ASV’s abundance per each dissection method. Black lines indicate the predicted relationship taken directly from the logistic regression models.

We ran a similar analysis pipeline for each extraction kit producing three total linear models (Supplemental Table 9). Similar to the dissection methods models, a relatively small fraction of the microbiome (25 ASVs) significantly predicts B*a*P exposure based on their abundance. One ASV, ASV00046 assigned to the Faecalibacterium genus, significantly predicted B*a*P exposure across all three extraction kits (its abundance is positively associated with B*a*P exposure; Supplemental Fig. 8 and Supplemental Table 9). Three other ASVs significantly predicted B*a*P exposure across two extraction kit data sets, the above mentioned ASV0032, along with ASV00038 (Candidatus_Finniella) and ASV00133 (Lachnospiraceae_Genus), all of which are positively associated with B*a*P exposure (Fig. 7; Supplemental Fig. 8; Supplemental Table 9). These results indicate that efforts to identify specific microbes that link to environmental chemical exposure may be impacted by the specific DNA extraction kit used in the investigation.

## Discussion

Our investigation of nearly 400 zebrafish larvae, half of which were exposed to 10 µM B*a*P, clarifies how various experimental decisions can affect the outcome of studies that investigate the effects of environmental chemical exposure on gut microbiome assembly in the zebrafish model. In particular, our study design allowed us to rigorously assess the effects of dissection method, DNA extraction kit, and single versus triplicate PCR. From these results, we find that measures of the zebrafish gut microbiome are generally robust to single versus triplicate PCR, though there is some indication that single PCR may result in different imputations of the composition of the rare biosphere. This is consistent with the rationale underlying triplicate PCR—stochastic effects are likely to play a larger role in the amplification of rare templates during PCR. This effect may or may or may not present complications for studies depending on their objectives. Moreover, this effect may matter less than the inclusion of additional biological replicates and, when in conflict, studies may prefer to prioritize biological replicates over technical PCR replicates.

Additionally, we find that dissection methods and DNA extraction kits have a relatively large impact on the inference of gut microbiome biodiversity and composition. In fact, these variables generally elicited more of an effect on the inferred alpha-diversity and beta-diversity of the gut microbiome than did exposure to B*a*P. Despite this fact, all methods revealed an effect of B*a*P on the composition of the gut microbiome. These methods also all uncovered B*a*P effects on the alpha-diversity of the gut microbiome, with the exception of when Nucleospin DNA extraction kits were applied to dissected intestinal tissues. Overall, these results indicate that different dissection methods and DNA extraction kits can be used to detect the effect of exposure on the gut microbiome, at least in the case of B*a*P, though caution may need to be applied when analyses hinge on precise estimates of community compositions and especially alpha-diversity. Moreover, researchers attempting to integrate data from studies that implemented different approaches will need to carefully consider the effect of the different approaches on the interpretation of the results. This point is underscored in our phylotype-level analyses, which identify very few ASVs that are consistently linked to exposure across methods. In fact, only one ASV, ASV0032, was a consistent indicator across dissection methods, though it was congruent between methods. Analyses examining phylotypes at higher taxonomic orders (LEfSe; genus through phylum) revealed indicators that were more consistent across methods. Such results imply that aggregating ASVs (or ESVs, OTUs, or ZOTUs) into higher taxonomic levels or phylogenetic units^56^ may be required for direct comparisons of the effects of toxicant exposures across studies. Overall, our observations indicate that when using zebrafish to screen for exposure impacts on microbiome composition as a whole (e.g. for rapidly screening for effects of different toxicants), it may be sufficient to use lower cost, higher scale approaches, such as using whole fish rather than extracted intestines to sample microbiomes, inexpensive kits to extract DNA, and using single PCR to prepare 16S libraries. Whereas, for research focusing on the effects of toxicant exposure on specific microbial phylotypes, implementation of more rigorous, lower throughput, and more expensive approaches may be necessary. At the very least, it is important to standardize methods across studies when integration of data across these studies is critical to the experimental objectives.

There are, of course, a number of caveats to the results presented here. First, we were only able to consider a single toxicant: B*a*P. While B*a*P exhibits a measurable and statistically significant effect on the composition and diversity of the larval zebrafish gut microbiome, it does not do so in an as overwhelming a manner as an antibiotic^6^ or more acute toxicant might. In such cases, the effects of dissection method, PCR replicates, or extraction kit on results may be completely overpowered by the effect of the treatment. Conversely, for toxicants (or exposure concentrations) that may have an even more subtle effect on the larval zebrafish gut microbiome than 10 µM B*a*P, greater consideration should be given to which methods to utilize, especially given that the microbiome’s sensitivity to exposure may be dose dependent. Likewise, these methods were only tested in larval zebrafish. These results may not necessarily translate to later zebrafish life stages, especially given that exposure may elicit different impacts on the gut microbiome as a function of the developmental timing of exposure. Moreover, our findings are principally pertinent to the zebrafish model system, and similar work should be conducted using other model systems to determine the best set of methods for each. Lastly, since there is no ground truth data about the effect of B*a*P on the zebrafish gut microbiome, these results do not tell us which methods give the most accurate results. Rather, our analyses enable us to measure the effect of different methodological decisions on the ability to discriminate between larval zebrafish microbiomes that have been exposed to B*a*P and those that have not. That said, our observations indicate that embryonic exposure to 10 µM B*a*P impacts the assembly of the gut microbiome given that almost all experimental parameters we explored revealed B*a*P exposure effects on alpha- and beta-diversity. This apparent effect of B*a*P on microbiome assembly adds to and is consistent with prior work that documented the gut microbiome’s sensitivity to B*a*P at alternative stages of life in other host taxa, including juvenile Tilapia^57^, adult^58^ and juvenile^59^ fathead minnows, scallops^60^, and adult mice^61^. While these collective results indicate that B*a*P is capable of eliciting measurable effects on the gut microbiome, future work may endeavor to test these methods for accuracy utilizing gnotobiotic models with a defined microbiome input.

In conclusion, we have shown that regardless of the dissection method, PCR method, or DNA extraction kit we analyzed, we were able to discern a statistically significant effect of 10 µM B*a*P exposure on the composition of the gut microbiome of larval zebrafish. However, the interpretation of this exposure effect, and in particular which phylotypes are shown to be most affected by exposure, is influenced by the particular set of methods we applied to each sample. Researchers utilizing the larval zebrafish model for microbiome studies will hopefully find herein helpful guidance in designing their own experiments, or for comparing the results from different studies within the field.

## Supplementary Information

Supplementary Information.

## Data Availability

Raw sequences are available in the NCBI short read archive under the BioProject PRJNA823740 (https://www.ncbi.nlm.nih.gov/bioproject/PRJNA823740). Code for analyses can be accessed at https://github.com/kstagaman/stagaman_etal_2022.
