# Supplementary material for: Experimental methods modestly impact interpretation of the effect of environmental exposures on the larval zebrafish gut microbiome
Source: Sci Rep. 2022 Aug 25;12:14538. doi: 10.1038/s41598-022-18532-x (PMC9411601; doi:10.1038/s41598-022-18532-x)
Supplement: Supplementary file 1 — Supplementary Information. [file 41598_2022_18532_MOESM1_ESM.pdf]

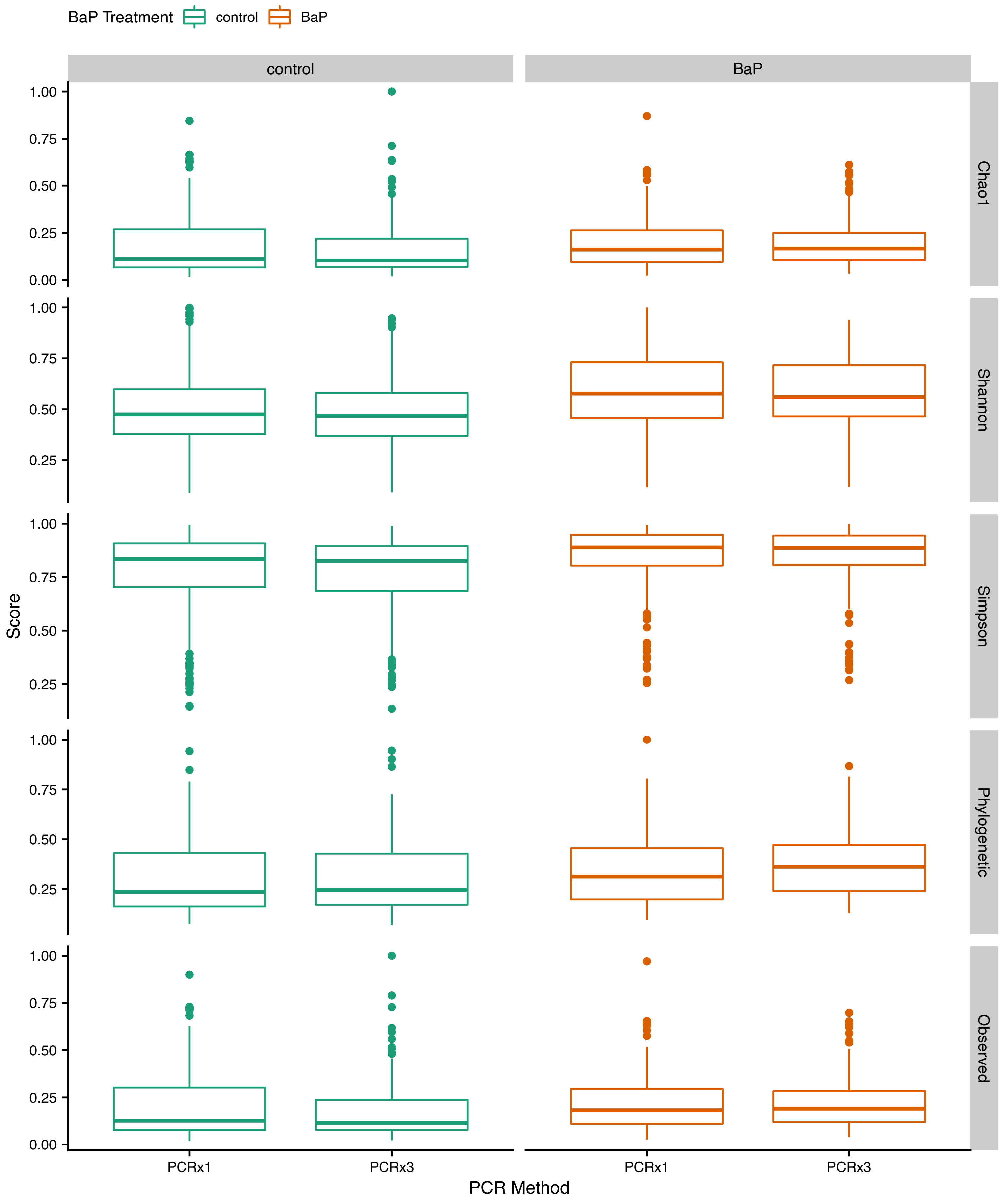

BaP Treatment
control
BaP
control
BaP
Chao1
Shannon
Simpson
Phylogenetic
Observed
Score
1.00
0.75
0.50
0.25
0.00
PCRx1
PCRx3
PCR Method

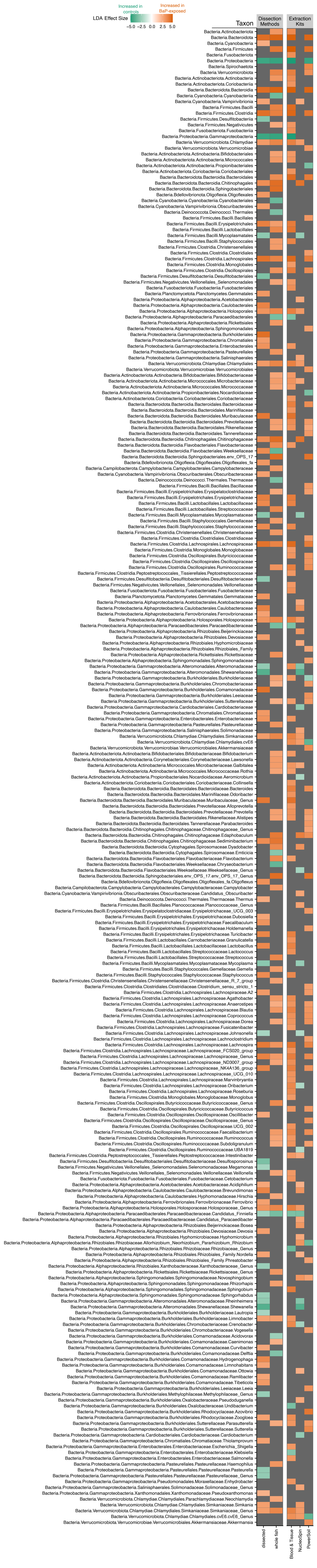
Increased in controls
Increased in BaP-exposed
LDA Effect Size
−5.0 −2.5 0.0 2.5 5.0
Taxon
Dissection Methods
Extraction Kits
dissected
whole fish
Blood & Tissue
NucleoSpin
PowerSoil

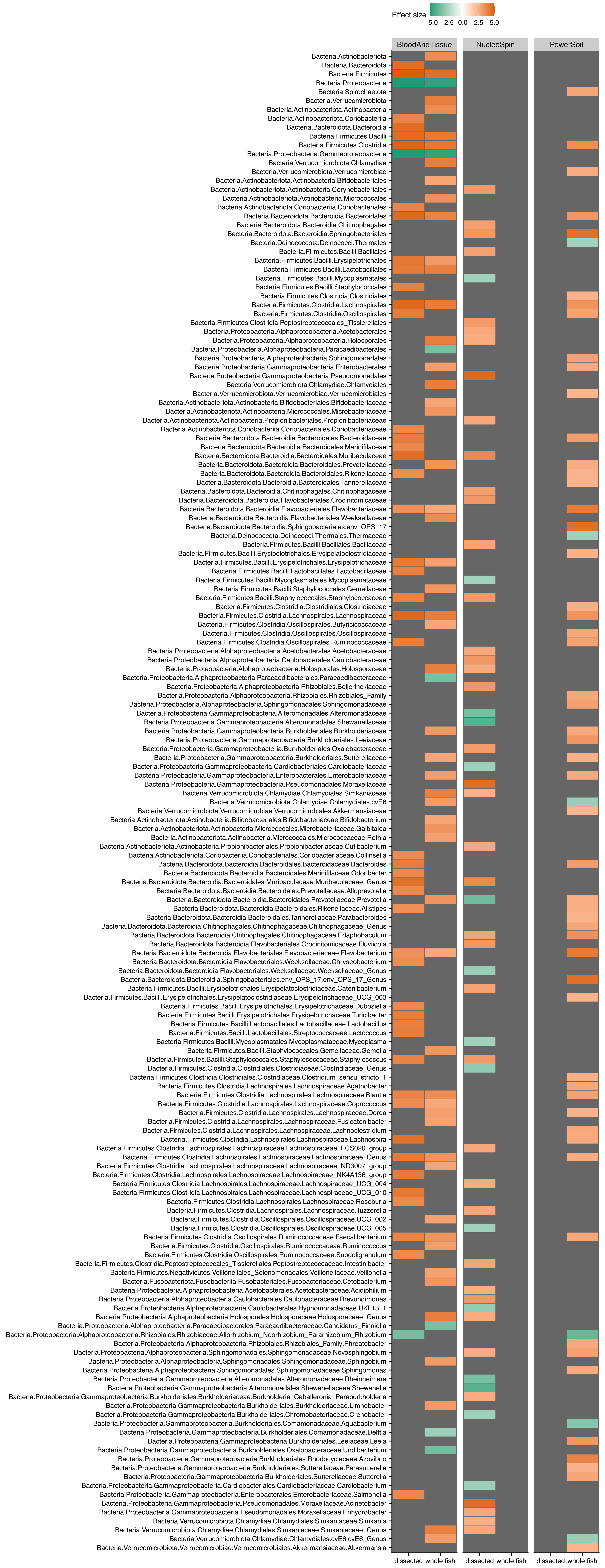
Effect size
−5.0 −2.5 0.0 2.5 5.0
BloodAndTissue
NucleoSpin
PowerSoil
Bacteria.Actinobacteriota
Bacteria.Bacteroidota
Bacteria.Firmicutes
Bacteria.Proteobacteria
Bacteria.Spirochaetota
Bacteria.Verrucomicrobiota
Bacteria.Actinobacteriota.Actinobacteria
Bacteria.Actinobacteriota.Coriobacteriia
Bacteria.Bacteroidota.Bacteroidia
Bacteria.Firmicutes.Bacilli
Bacteria.Firmicutes.Clostridia
Bacteria.Proteobacteria.Gammaproteobacteria
Bacteria.Verrucomicrobiota.Chlamydiae
Bacteria.Verrucomicrobiota.Verrucomicrobiae
Bacteria.Actinobacteriota.Actinobacteria.Bifidobacteriales
Bacteria.Actinobacteriota.Actinobacteria.Corynebacteriales
Bacteria.Actinobacteriota.Actinobacteria.Micrococcales
Bacteria.Actinobacteriota.Coriobacteriia.Coriobacteriales
Bacteria.Bacteroidota.Bacteroidia.Bacteroidales
Bacteria.Bacteroidota.Bacteroidia.Chitinophagales
Bacteria.Bacteroidota.Bacteroidia.Sphingobacteriales
Bacteria.Deinococcota.Deinococci.Thermales
Bacteria.Firmicutes.Bacilli.Bacillales
Bacteria.Firmicutes.Bacilli.Erysipelotrichales
Bacteria.Firmicutes.Bacilli.Lactobacillales
Bacteria.Firmicutes.Bacilli.Mycoplasmatales
Bacteria.Firmicutes.Bacilli.Staphylococcales
Bacteria.Firmicutes.Clostridia.Clostridiales
Bacteria.Firmicutes.Clostridia.Lachnospirales
Bacteria.Firmicutes.Clostridia.Oscillospirales
Bacteria.Firmicutes.Clostridia.Peptostreptococcales_Tissierellales
Bacteria.Proteobacteria.Alphaproteobacteria.Acetobacterales
Bacteria.Proteobacteria.Alphaproteobacteria.Holosporales
Bacteria.Proteobacteria.Alphaproteobacteria.Paracaedibacterales
Bacteria.Proteobacteria.Alphaproteobacteria.Sphingomonadales
Bacteria.Proteobacteria.Gammaproteobacteria.Enterobacterales
Bacteria.Proteobacteria.Gammaproteobacteria.Pseudomonadales
Bacteria.Verrucomicrobiota.Chlamydiae.Chlamydiales
Bacteria.Verrucomicrobiota.Verrucomicrobiae.Verrucomicrobiales
Bacteria.Actinobacteriota.Actinobacteria.Bifidobacteriales.Bifidobacteriaceae
Bacteria.Actinobacteriota.Actinobacteria.Micrococcales.Microbacteriaceae
Bacteria.Actinobacteriota.Actinobacteria.Propionibacteriales.Propionibacteriaceae
Bacteria.Actinobacteriota.Coriobacteriia.Coriobacteriales.Coriobacteriaceae
Bacteria.Bacteroidota.Bacteroidia.Bacteroidales.Bacteroidaceae
Bacteria.Bacteroidota.Bacteroidia.Bacteroidales.Marinifilaceae
Bacteria.Bacteroidota.Bacteroidia.Bacteroidales.Muribaculaceae
Bacteria.Bacteroidota.Bacteroidia.Bacteroidales.Prevotellaceae
Bacteria.Bacteroidota.Bacteroidia.Bacteroidales.Rikenellaceae
Bacteria.Bacteroidota.Bacteroidia.Bacteroidales.Tannerellaceae
Bacteria.Bacteroidota.Bacteroidia.Chitinophagales.Chitinophagaceae
Bacteria.Bacteroidota.Bacteroidia.Flavobacteriales.Crocinitomicaceae
Bacteria.Bacteroidota.Bacteroidia.Flavobacteriales.Flavobacteriaceae
Bacteria.Bacteroidota.Bacteroidia.Flavobacteriales.Weeksellaceae
Bacteria.Bacteroidota.Bacteroidia.Sphingobacteriales.env_OPS_17
Bacteria.Deinococcota.Deinococci.Thermales.Thermaceae
Bacteria.Firmicutes.Bacilli.Bacillales.Bacillaceae
Bacteria.Firmicutes.Bacilli.Erysipelotrichales.Erysipelatoclostridiaceae
Bacteria.Firmicutes.Bacilli.Erysipelotrichales.Erysipelotrichaceae
Bacteria.Firmicutes.Bacilli.Lactobacillales.Lactobacillaceae
Bacteria.Firmicutes.Bacilli.Mycoplasmatales.Mycoplasmataceae
Bacteria.Firmicutes.Bacilli.Staphylococcales.Gemellaceae
Bacteria.Firmicutes.Bacilli.Staphylococcales.Staphylococcaceae
Bacteria.Firmicutes.Clostridia.Clostridiales.Clostridiaceae
Bacteria.Firmicutes.Clostridia.Lachnospirales.Lachnospiraceae
Bacteria.Firmicutes.Clostridia.Oscillospirales.Butyricicoccaceae
Bacteria.Firmicutes.Clostridia.Oscillospirales.Oscillospiraceae
Bacteria.Firmicutes.Clostridia.Oscillospirales.Ruminococcaceae
Bacteria.Proteobacteria.Alphaproteobacteria.Acetobacterales.Acetobacteraceae
Bacteria.Proteobacteria.Alphaproteobacteria.Caulobacterales.Caulobacteraceae
Bacteria.Proteobacteria.Alphaproteobacteria.Holosporales.Holosporaceae
Bacteria.Proteobacteria.Alphaproteobacteria.Paracaedibacterales.Paracaedibacteraceae
Bacteria.Proteobacteria.Alphaproteobacteria.Rhizobiales.Beijerinckiaceae
Bacteria.Proteobacteria.Alphaproteobacteria.Rhizobiales.Rhizobiales_Family
Bacteria.Proteobacteria.Alphaproteobacteria.Sphingomonadales.Sphingomonadaceae
Bacteria.Proteobacteria.Gammaproteobacteria.Alteromonadales.Alteromonadaceae
Bacteria.Proteobacteria.Gammaproteobacteria.Alteromonadales.Shewanellaceae
Bacteria.Proteobacteria.Gammaproteobacteria.Burkholderiales.Burkholderiaceae
Bacteria.Proteobacteria.Gammaproteobacteria.Burkholderiales.Leeiaceae
Bacteria.Proteobacteria.Gammaproteobacteria.Burkholderiales.Oxalobacteraceae
Bacteria.Proteobacteria.Gammaproteobacteria.Burkholderiales.Sutterellaceae
Bacteria.Proteobacteria.Gammaproteobacteria.Cardiobacteriales.Cardiobacteriaceae
Bacteria.Proteobacteria.Gammaproteobacteria.Enterobacterales.Enterobacteriaceae
Bacteria.Proteobacteria.Gammaproteobacteria.Pseudomonadales.Moraxellaceae
Bacteria.Verrucomicrobiota.Chlamydiae.Chlamydiales.Simkaniaceae
Bacteria.Verrucomicrobiota.Chlamydiae.Chlamydiales.cvE6
Bacteria.Verrucomicrobiota.Verrucomicrobiae.Verrucomicrobiales.Akkermansiaceae
Bacteria.Actinobacteriota.Actinobacteria.Bifidobacteriales.Bifidobacteriaceae.Bifidobacterium
Bacteria.Actinobacteriota.Actinobacteria.Micrococcales.Microbacteriaceae.Galbitalea
Bacteria.Actinobacteriota.Actinobacteria.Micrococcales.Micrococcaceae.Rothia
Bacteria.Actinobacteriota.Actinobacteria.Propionibacteriales.Propionibacteriaceae.Cutibacterium
Bacteria.Actinobacteriota.Coriobacteriia.Coriobacteriales.Coriobacteriaceae.Collinsella
Bacteria.Bacteroidota.Bacteroidia.Bacteroidales.Bacteroidaceae.Bacteroides
Bacteria.Bacteroidota.Bacteroidia.Bacteroidales.Marinifilaceae.Odoribacter
Bacteria.Bacteroidota.Bacteroidia.Bacteroidales.Muribaculaceae.Muribaculaceae_Genus
Bacteria.Bacteroidota.Bacteroidia.Bacteroidales.Prevotellaceae.Alloprevotella
Bacteria.Bacteroidota.Bacteroidia.Bacteroidales.Prevotellaceae.Prevotella
Bacteria.Bacteroidota.Bacteroidia.Bacteroidales.Rikenellaceae.Alistipes
Bacteria.Bacteroidota.Bacteroidia.Bacteroidales.Tannerellaceae.Parabacteroides
Bacteria.Bacteroidota.Bacteroidia.Chitinophagales.Chitinophagaceae.Chitinophagaceae_Genus
Bacteria.Bacteroidota.Bacteroidia.Chitinophagales.Chitinophagaceae.Edaphobaculum
Bacteria.Bacteroidota.Bacteroidia.Flavobacteriales.Crocinitomicaceae.Fluviicola
Bacteria.Bacteroidota.Bacteroidia.Flavobacteriales.Flavobacteriaceae.Flavobacterium
Bacteria.Bacteroidota.Bacteroidia.Flavobacteriales.Weeksellaceae.Chryseobacterium
Bacteria.Bacteroidota.Bacteroidia.Flavobacteriales.Weeksellaceae.Weeksellaceae_Genus
Bacteria.Bacteroidota.Bacteroidia.Sphingobacteriales.env_OPS_17.env_OPS_17_Genus
Bacteria.Firmicutes.Bacilli.Erysipelotrichales.Erysipelatoclostridiaceae.Catenibacterium
Bacteria.Firmicutes.Bacilli.Erysipelotrichales.Erysipelatoclostridiaceae.Erysipelotrichaceae_UCG_003
Bacteria.Firmicutes.Bacilli.Erysipelotrichales.Erysipelotrichaceae.Dubosiella
Bacteria.Firmicutes.Bacilli.Erysipelotrichales.Erysipelotrichaceae.Turicibacter
Bacteria.Firmicutes.Bacilli.Lactobacillales.Lactobacillaceae.Lactobacillus
Bacteria.Firmicutes.Bacilli.Lactobacillales.Streptococcaceae.Lactococcus
Bacteria.Firmicutes.Bacilli.Mycoplasmatales.Mycoplasmataceae.Mycoplasma
Bacteria.Firmicutes.Bacilli.Staphylococcales.Gemellaceae.Gemella
Bacteria.Firmicutes.Bacilli.Staphylococcales.Staphylococcaceae.Staphylococcus
Bacteria.Firmicutes.Clostridia.Clostridiales.Clostridiaceae.Clostridiaceae_Genus
Bacteria.Firmicutes.Clostridia.Clostridiales.Clostridiaceae.Clostridium_sensu_stricto_1
Bacteria.Firmicutes.Clostridia.Lachnospirales.Lachnospiraceae.Agathobacter
Bacteria.Firmicutes.Clostridia.Lachnospirales.Lachnospiraceae.Blautia
Bacteria.Firmicutes.Clostridia.Lachnospirales.Lachnospiraceae.Coprococcus
Bacteria.Firmicutes.Clostridia.Lachnospirales.Lachnospiraceae.Dorea
Bacteria.Firmicutes.Clostridia.Lachnospirales.Lachnospiraceae.Fusicatenibacter
Bacteria.Firmicutes.Clostridia.Lachnospirales.Lachnospiraceae.Lachnoclostridium
Bacteria.Firmicutes.Clostridia.Lachnospirales.Lachnospiraceae.Lachnospira
Bacteria.Firmicutes.Clostridia.Lachnospirales.Lachnospiraceae.Lachnospiraceae_FCS020_group
Bacteria.Firmicutes.Clostridia.Lachnospirales.Lachnospiraceae.Lachnospiraceae_Genus
Bacteria.Firmicutes.Clostridia.Lachnospirales.Lachnospiraceae.Lachnospiraceae_ND3007_group
Bacteria.Firmicutes.Clostridia.Lachnospirales.Lachnospiraceae.Lachnospiraceae_NK4A136_group
Bacteria.Firmicutes.Clostridia.Lachnospirales.Lachnospiraceae.Lachnospiraceae_UCG_004
Bacteria.Firmicutes.Clostridia.Lachnospirales.Lachnospiraceae.Lachnospiraceae_UCG_010
Bacteria.Firmicutes.Clostridia.Lachnospirales.Lachnospiraceae.Roseburia
Bacteria.Firmicutes.Clostridia.Lachnospirales.Lachnospiraceae.Tuzzerella
Bacteria.Firmicutes.Clostridia.Oscillospirales.Oscillospiraceae.UCG_002
Bacteria.Firmicutes.Clostridia.Oscillospirales.Oscillospiraceae.UCG_005
Bacteria.Firmicutes.Clostridia.Oscillospirales.Ruminococcaceae.Faecalibacterium
Bacteria.Firmicutes.Clostridia.Oscillospirales.Ruminococcaceae.Ruminococcus
Bacteria.Firmicutes.Clostridia.Oscillospirales.Ruminococcaceae.Subdoligranulum
Bacteria.Firmicutes.Clostridia.Peptostreptococcales_Tissierellales.Peptostreptococcaceae.Intestinibacter
Bacteria.Firmicutes.Negativicutes.Veillonellales_Selenomonadales.Veillonellaceae.Veillonella
Bacteria.Fusobacteriota.Fusobacteriia.Fusobacteriales.Fusobacteriaceae.Cetobacterium
Bacteria.Proteobacteria.Alphaproteobacteria.Acetobacterales.Acetobacteraceae.Acidiphilium
Bacteria.Proteobacteria.Alphaproteobacteria.Caulobacterales.Caulobacteraceae.Brevundimonas
Bacteria.Proteobacteria.Alphaproteobacteria.Caulobacterales.Hyphomonadaceae.UKL13_1
Bacteria.Proteobacteria.Alphaproteobacteria.Holosporales.Holosporaceae.Holosporaceae_Genus
Bacteria.Proteobacteria.Alphaproteobacteria.Paracaedibacterales.Paracaedibacteraceae.Candidatus_Finniella
Bacteria.Proteobacteria.Alphaproteobacteria.Rhizobiales.Rhizobiaceae.Allorhizobium_Neorhizobium_Pararhizobium_Rhizobium
Bacteria.Proteobacteria.Alphaproteobacteria.Rhizobiales.Rhizobiales_Family.Phreatobacter
Bacteria.Proteobacteria.Alphaproteobacteria.Sphingomonadales.Sphingomonadaceae.Novosphingobium
Bacteria.Proteobacteria.Alphaproteobacteria.Sphingomonadales.Sphingomonadaceae.Sphingobium
Bacteria.Proteobacteria.Alphaproteobacteria.Sphingomonadales.Sphingomonadaceae.Sphingomonas
Bacteria.Proteobacteria.Gammaproteobacteria.Alteromonadales.Alteromonadaceae.Rheinheimera
Bacteria.Proteobacteria.Gammaproteobacteria.Alteromonadales.Shewanellaceae.Shewanella
Bacteria.Proteobacteria.Gammaproteobacteria.Burkholderiales.Burkholderiaceae.Burkholderia_Caballeronia_Paraburkholderia
Bacteria.Proteobacteria.Gammaproteobacteria.Burkholderiales.Burkholderiaceae.Limnobacter
Bacteria.Proteobacteria.Gammaproteobacteria.Burkholderiales.Chromobacteriaceae.Crenobacter
Bacteria.Proteobacteria.Gammaproteobacteria.Burkholderiales.Comamonadaceae.Aquabacterium
Bacteria.Proteobacteria.Gammaproteobacteria.Burkholderiales.Comamonadaceae.Delftia
Bacteria.Proteobacteria.Gammaproteobacteria.Burkholderiales.Leeiaceae.Leeia
Bacteria.Proteobacteria.Gammaproteobacteria.Burkholderiales.Oxalobacteraceae.Undibacterium
Bacteria.Proteobacteria.Gammaproteobacteria.Burkholderiales.Rhodocyclaceae.Azovibrio
Bacteria.Proteobacteria.Gammaproteobacteria.Burkholderiales.Sutterellaceae.Parasutterella
Bacteria.Proteobacteria.Gammaproteobacteria.Burkholderiales.Sutterellaceae.Sutterella
Bacteria.Proteobacteria.Gammaproteobacteria.Cardiobacteriales.Cardiobacteriaceae.Cardiobacterium
Bacteria.Proteobacteria.Gammaproteobacteria.Enterobacterales.Enterobacteriaceae.Salmonella
Bacteria.Proteobacteria.Gammaproteobacteria.Pseudomonadales.Moraxellaceae.Acinetobacter
Bacteria.Proteobacteria.Gammaproteobacteria.Pseudomonadales.Moraxellaceae.Enhydrobacter
Bacteria.Verrucomicrobiota.Chlamydiae.Chlamydiales.Simkaniaceae.Simkania
Bacteria.Verrucomicrobiota.Chlamydiae.Chlamydiales.Simkaniaceae.Simkaniaceae_Genus
Bacteria.Verrucomicrobiota.Chlamydiae.Chlamydiales.cvE6.cvE6_Genus
Bacteria.Verrucomicrobiota.Verrucomicrobiae.Verrucomicrobiales.Akkermansiaceae.Akkermansia
dissected whole fish
dissected whole fish
dissected whole fish

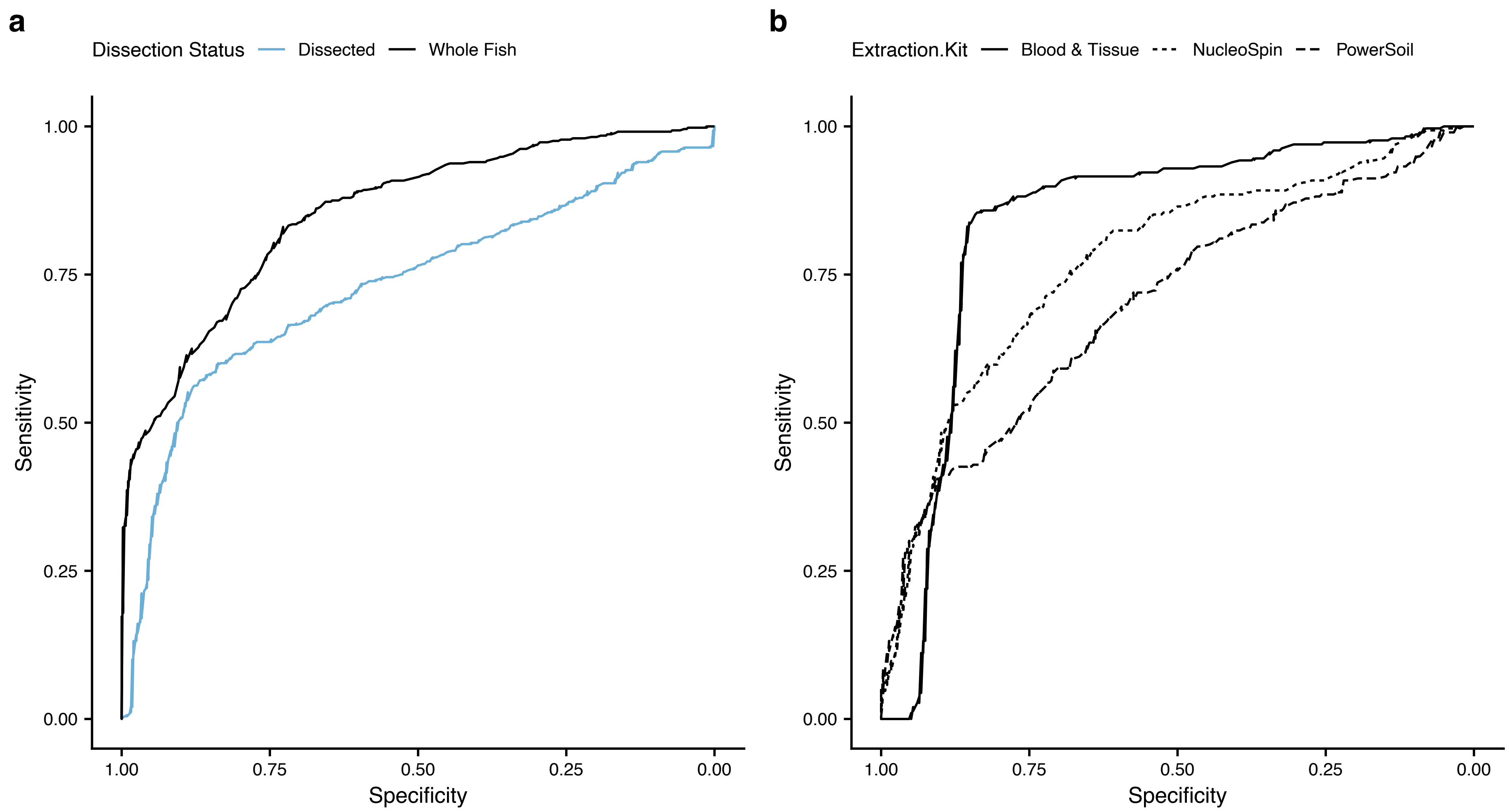


| Dissection Method | AUC |
|---|---|
| Dissected | 0.730 |
| Whole Fish | 0.854 |

| Extraction Kit | AUC |
|---|---|
| Blood & Tissue | 0.838 |
| NucleoSpin | 0.773 |
| PowerSoil | 0.701 |

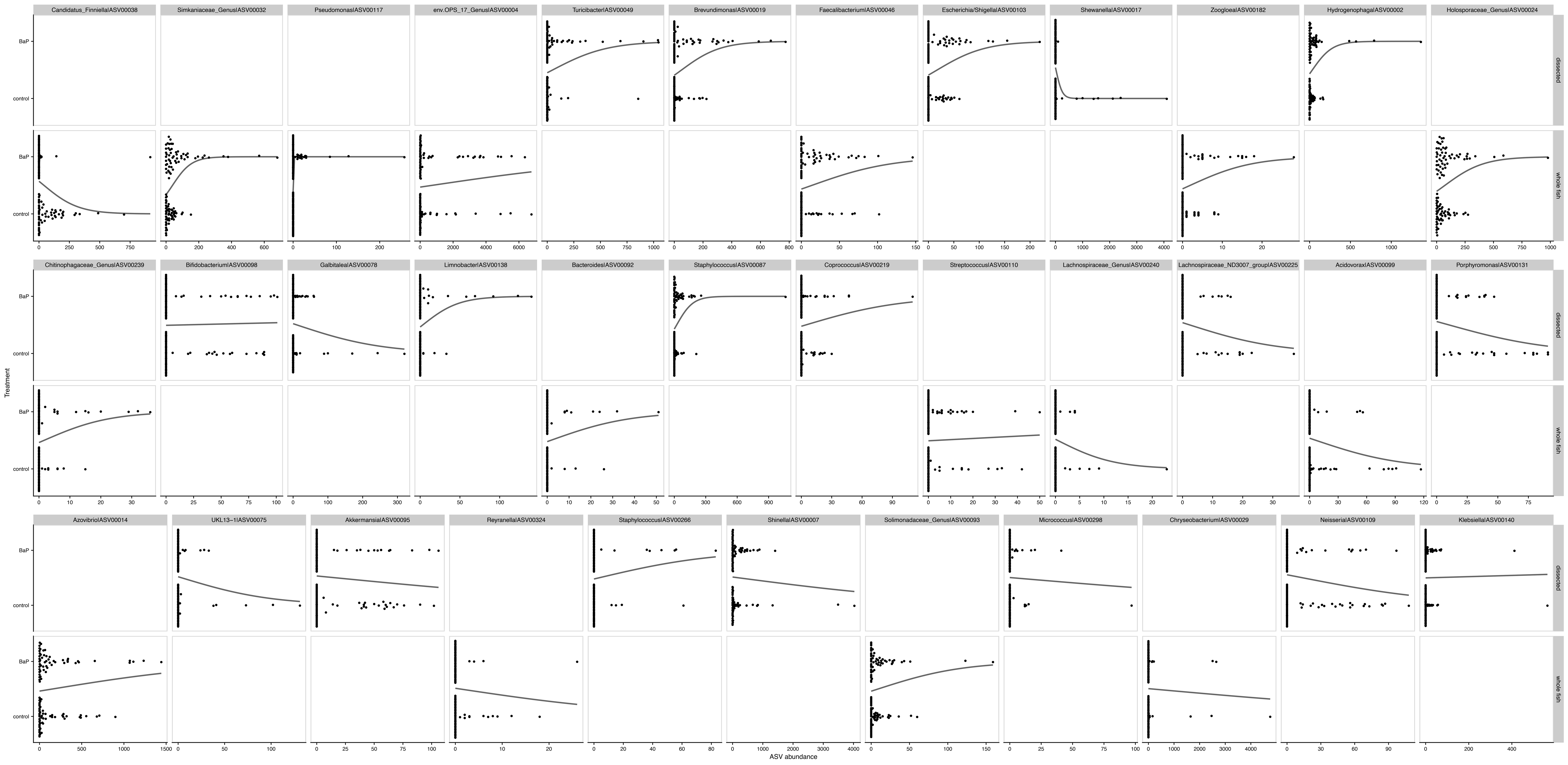
Candidatus_Finniella|ASV00038
Simkaniaceae_Genus|ASV00032
Pseudomonas|ASV00117
env.OPS_17_Genus|ASV00004
Turicibacter|ASV00049
Brevundimonas|ASV00019
Faecalibacterium|ASV00046
Escherichia/Shigella|ASV00103
Shewanella|ASV00017
Zoogloea|ASV00182
Hydrogenophaga|ASV00002
Holosporaceae_Genus|ASV00024
Chitinophagaceae_Genus|ASV00239
Bifidobacterium|ASV00098
Galbitalea|ASV00078
Limnobacter|ASV00138
Bacteroides|ASV00092
Staphylococcus|ASV00087
Coprococcus|ASV00219
Streptococcus|ASV00110
Lachnospiraceae_Genus|ASV00240
Lachnospiraceae_ND3007_group|ASV00225
Acidovorax|ASV00099
Porphyromonas|ASV00131
Azovibrio|ASV00014
UKL13-1|ASV00075
Akkermansia|ASV00095
Reyranella|ASV00324
Staphylococcus|ASV00266
Shinella|ASV00007
Solimonadaceae_Genus|ASV00093
Micrococcus|ASV00298
Chryseobacterium|ASV00029
Neisseria|ASV00109
Klebsiella|ASV00140
BaP
control
dissected
whole fish
Treatment
ASV abundance

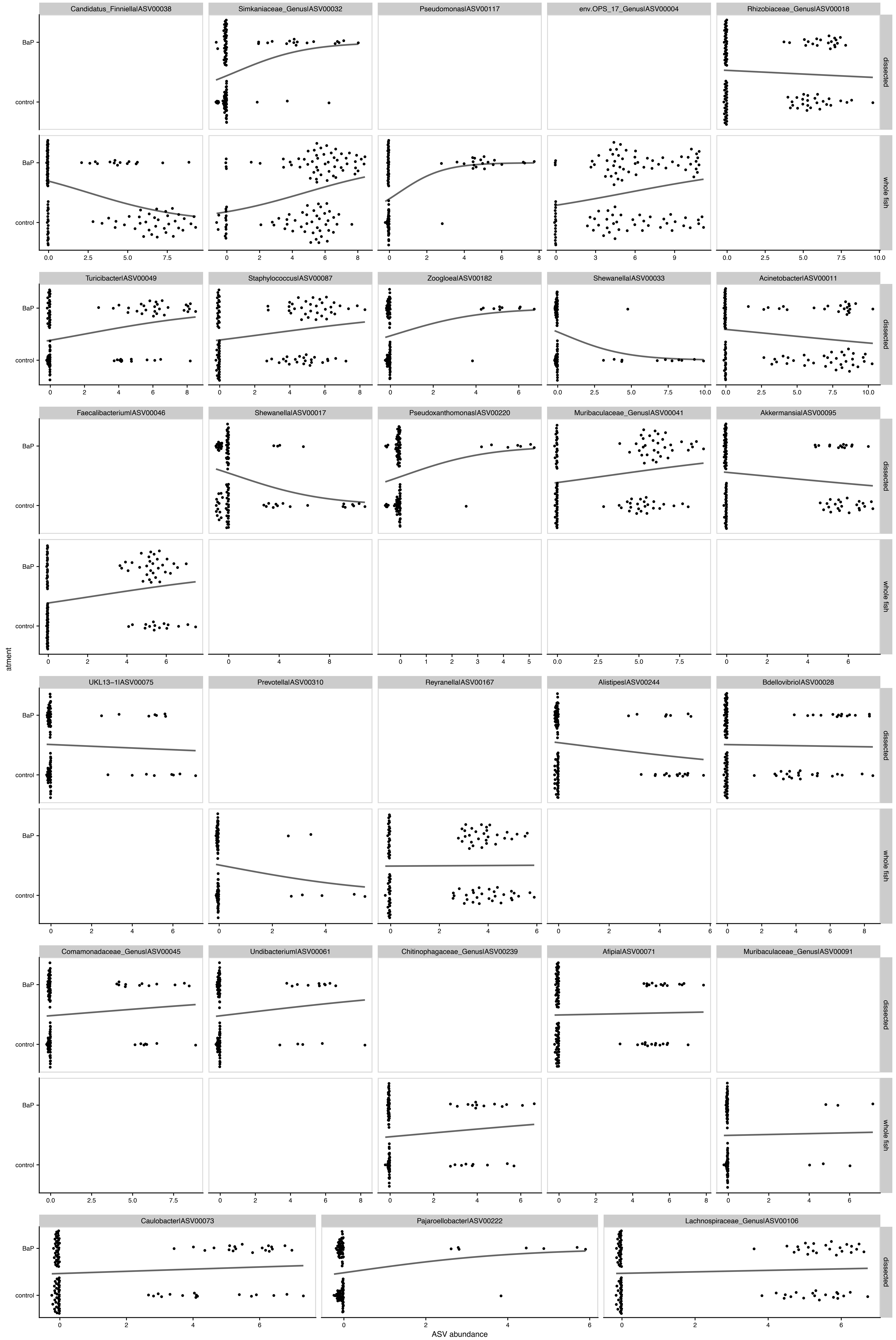

Candidatus_Finniella|ASV00038
Simkaniaceae_Genus|ASV00032
Pseudomonas|ASV00117
env.OPS_17_Genus|ASV00004
Rhizobiaceae_Genus|ASV00018
Turicibacter|ASV00049
Staphylococcus|ASV00087
Zoogloea|ASV00182
Shewanella|ASV00033
Acinetobacter|ASV00011
Faecalibacterium|ASV00046
Shewanella|ASV00017
Pseudoxanthomonas|ASV00220
Muribaculaceae_Genus|ASV00041
Akkermansia|ASV00095
UKL13-1|ASV00075
Prevotella|ASV00310
Reyranella|ASV00167
Alistipes|ASV00244
Bdellovibrio|ASV00028
Comamonadaceae_Genus|ASV00045
Undibacterium|ASV00061
Chitinophagaceae_Genus|ASV00239
Afipia|ASV00071
Muribaculaceae_Genus|ASV00091
Caulobacter|ASV00073
Pajaroellobacter|ASV00222
Lachnospiraceae_Genus|ASV00106
dissected
whole fish
BaP
control
atment
ASV abundance

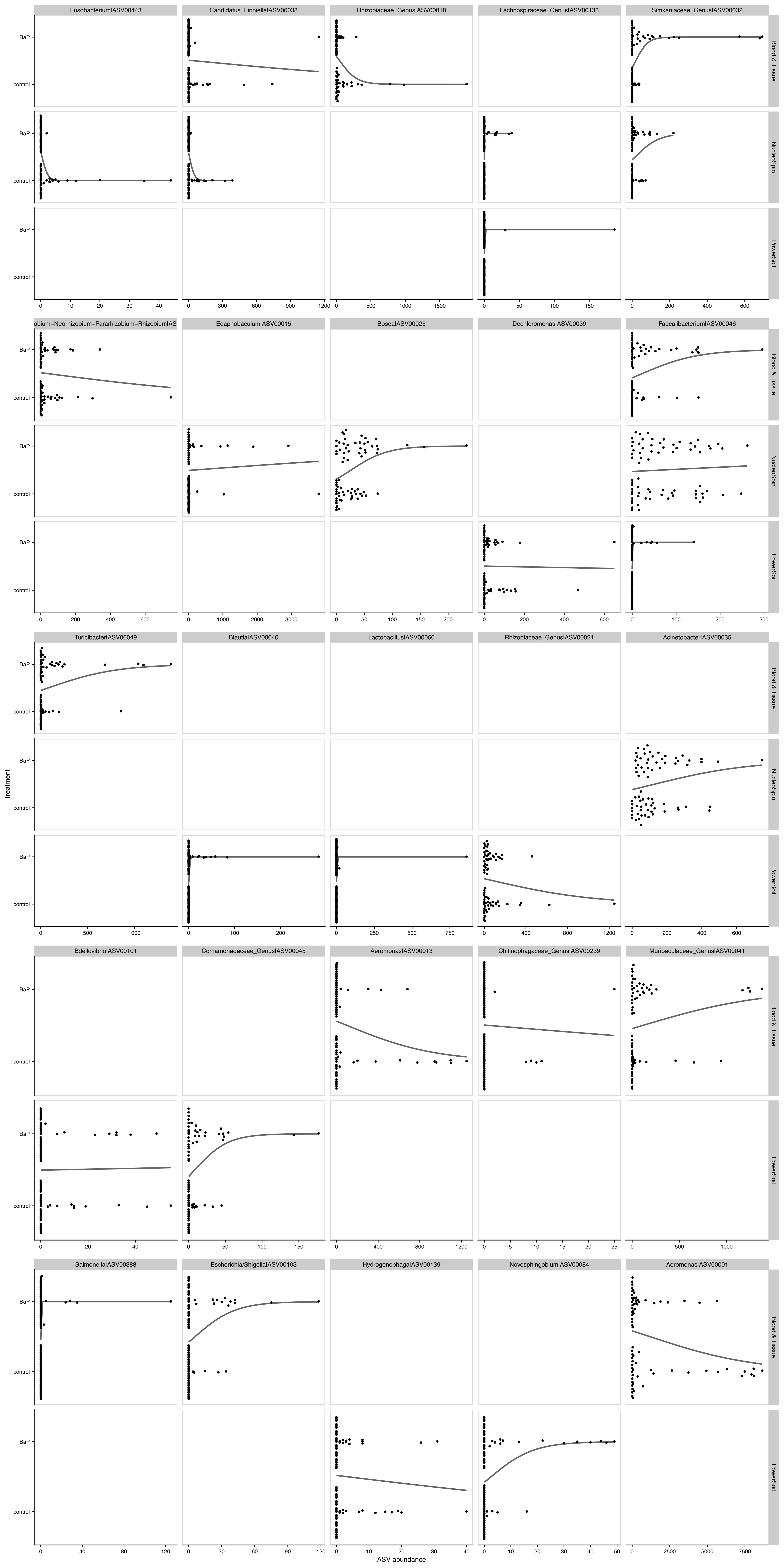
FusobacteriumlASV00443
Candidatus_FinniellalASV00038
Rhizobiaceae_GenuslASV00018
Lachnospiraceae_GenuslASV00133
Simkaniaceae_GenuslASV00032
EdaphobaculumlASV00015
BosealASV00025
DechloromonaslASV00039
FaecalibacteriumlASV00046
TuricibacterlASV00049
BlautialASV00040
LactobacilluslASV00060
Rhizobiaceae_GenuslASV00021
AcinetobacterlASV00035
BdellovibriolASV00101
Comamonadaceae_GenuslASV00045
AeromonaslASV00013
Chitinophagaceae_GenuslASV00239
Muribaculaceae_GenuslASV00041
SalmonellalASV00388
Escherichia/ShigellalASV00103
HydrogenophagalASV00139
NovosphingobiumlASV00084
AeromonaslASV00001
Blood & Tissue
NucleoSpin
PowerSoil
BaP
control
Treatment
ASV abundance

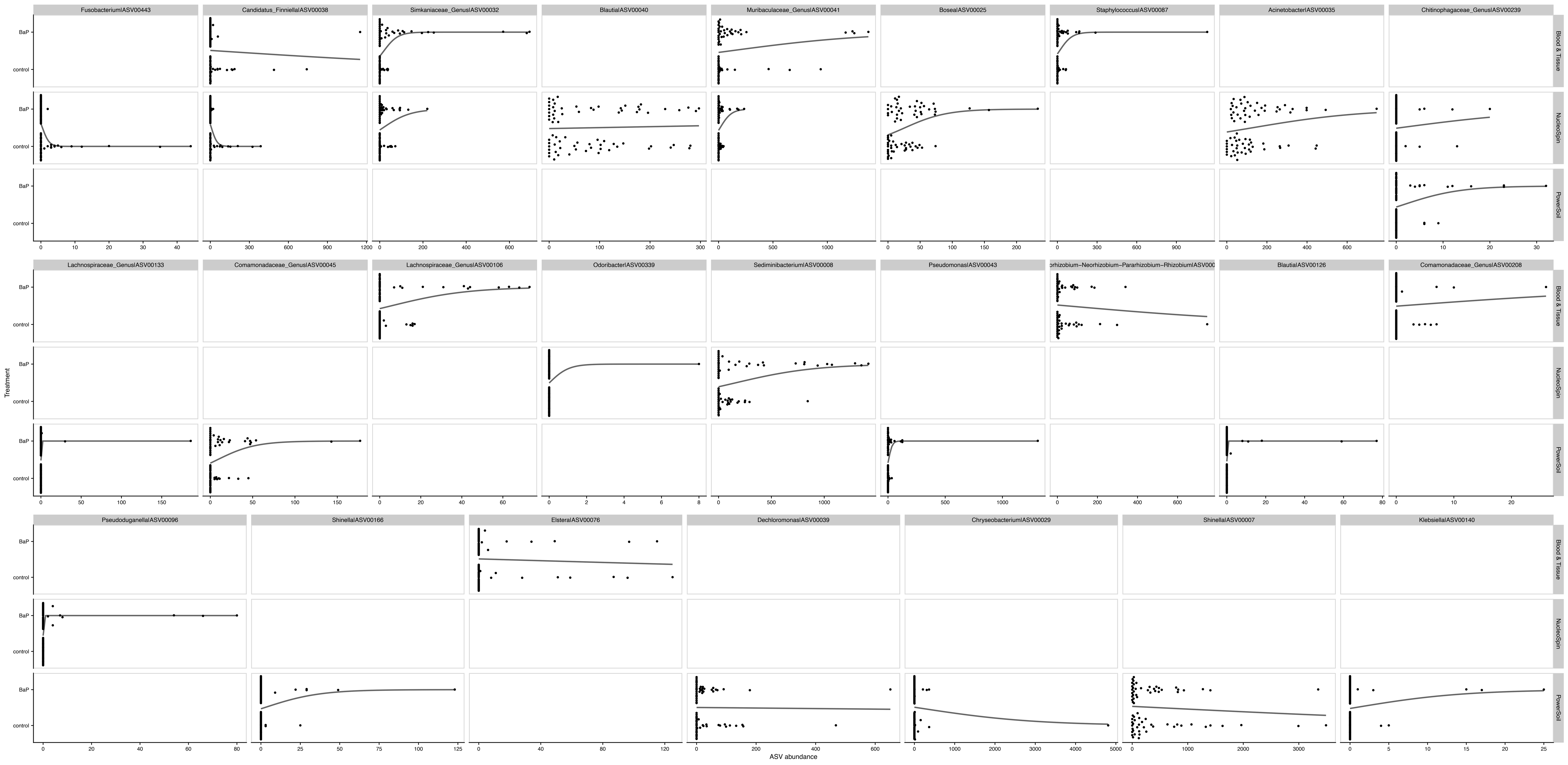
Fusobacterium|ASV00443
Candidatus_Finniella|ASV00038
Simkaniaceae_Genus|ASV00032
Blautia|ASV00040
Muribaculaceae_Genus|ASV00041
Bosea|ASV00025
Staphylococcus|ASV00087
Acinetobacter|ASV00035
Chitinophagaceae_Genus|ASV00239
Lachnospiraceae_Genus|ASV00133
Comamonadaceae_Genus|ASV00045
Lachnospiraceae_Genus|ASV00106
Odoribacter|ASV00339
Sediminibacterium|ASV00008
Pseudomonas|ASV00043
orhizobium−Neorhizobium−Pararhizobium−Rhizobium|ASV000
Blautia|ASV00126
Comamonadaceae_Genus|ASV00208
Pseudoduganella|ASV00096
Shinella|ASV00166
Elstera|ASV00076
Dechloromonas|ASV00039
Chryseobacterium|ASV00029
Shinella|ASV00007
Klebsiella|ASV00140
Blood & Tissue
NucleoSpin
PowerSoil
BaP
control
Treatment
ASV abundance

Supplemental Figure 1. Box plots of alpha-diversity metric scores by B*a*P exposure and PCR method (triplicate vs single reactions).

Supplemental Figure 2. Heatmap of statistically significant effect sizes estimated by LEfSe. This plot shows all phylotypes that were identified as indicators across any data set. The color of the square indicates the effect size with green meaning a phylotype is indicative of the control (no BaP exposure) while orange means a phylotype is indicative of BaP exposure.

Supplemental Figure 3. Receiver operating characteristic (ROC) curve plots for random forest models predicting BaP exposure from various data subsets, indicated by line color or line type, of rarefied ASV counts. (a) ROC curves for dissected gut versus whole fish, with the areas under the curve (AUCs) listed in the table below. (b) Similar data as in panel a, but for subsets by DNA extraction kit.

Supplemental Figure 4. Swarm plots of logistic regressions predicting BaP exposure from individual ASV abundances (rarefied) using data subsets partititioned by dissection method. ASVs are orded by importance from the preceding random forest analysis. Points indicate an ASV's abundance per each dissection method. Black lines indicate the predicted relationship taken directly from the logistic regression models.

Supplemental Figure 5. Swarm plots of logistic regressions predicting BaP exposure from individual ASV abundances (CLR-transformed) using data subsets partititioned by dissection method. ASVs are orded by importance from the preceding random forest analysis. (This first 8 ASVs in this figure comprised Figure 6). Points indicate an ASV's abundance per each dissection method. Black lines indicate the predicted relationship taken directly from the logistic regression models.

Supplemental Figure 6. Swarm plots of logistic regressions predicting BaP exposure from individual ASV abundances (rarefied) using data subsets partititioned by DNA extraction kit. ASVs are orded by importance from the preceding random forest analysis. Points indicate an ASV's abundance per each dissection method. Black lines indicate the predicted relationship taken directly from the logistic regression models.

Supplemental Figure 7. Swarm plots of logistic regressions predicting BaP exposure from individual ASV abundances (CLR-transformed) using data subsets partititioned by DNA extraction kit. ASVs are orded by importance from the preceding random forest analysis. (This first 8 ASVs in this figure comprised Figure 7). Points indicate an ASV's abundance per each dissection method. Black lines indicate the predicted relationship taken directly from the logistic regression models.

Supplemental Table 1. Counts of biological samples (so not including x3 and x1 PCR as separate samples) in each treatment set. We started with 384 (4 x 96) zebrafish embryos, and after dada2 processing (which include rejecting reads based on low quality) and rarefaction, we ended with 277 biological replicates. For analyses in which we tested the effects of dissection method and extraction kit together, we used the samples subsetted by extraction kit.

| BaP Treatment | Dissection Method | Extraction Kit | Count after QC | Count after subsampling |
|---|---|---|---|---|
| - | - | - | 277 | - |
| Unexposed | - | - | 123 | - |
| Exposed | - | - | 154 | - |
| - | Dissected | - | 127 | 112 |
| - | Whole fish | - | 150 | 112 |
| - | - | PowerSoil | 92 | 74 |
| - | - | NucleoSpin | 96 | 74 |
| - | - | Blood & Tissue | 89 | 74 |
| Unexposed | Dissected | - | 56 | 56 |
| Unexposed | Whole fish | - | 67 | 56 |
| Exposed | Dissected | - | 71 | 56 |
| Exposed | Whole fish | - | 83 | 56 |
| Unexposed | - | PowerSoil | 43 | 37 |
| Unexposed | - | NucleoSpin | 43 | 37 |
| Unexposed | - | Blood & Tissue | 37 | 37 |
| Exposed | - | PowerSoil | 49 | 37 |
| Exposed | - | NucleoSpin | 53 | 37 |
| Exposed | - | Blood & Tissue | 52 | 37 |
| Unexposed | Dissected | PowerSoil | 18 | 16 |
| Unexposed | Dissected | NucleoSpin | 22 | 20 |
| Unexposed | Dissected | Blood & Tissue | 16 | 16 |
| Unexposed | Whole fish | PowerSoil | 25 | 21 |
| Unexposed | Whole fish | NucleoSpin | 21 | 17 |
| Unexposed | Whole fish | Blood & Tissue | 21 | 21 |
| Exposed | Dissected | PowerSoil | 21 | 16 |
| Exposed | Dissected | NucleoSpin | 26 | 18 |
| Exposed | Dissected | Blood & Tissue | 24 | 18 |
| Exposed | Whole fish | PowerSoil | 28 | 21 |
| Exposed | Whole fish | NucleoSpin | 27 | 19 |
| Exposed | Whole fish | Blood & Tissue | 28 | 19 |

Supplemental Table 2. Alpha-diversity by PCR method MM-GLM results (n = 277)

| metric | term | statistic | df | p.value | sig |
|---|---|---|---|---|---|
| Chao1 | BaP Treatment | 0.59 | 1 | 0.444 | |
| | PCR Method | 0.08 | 1 | 0.783 | |
| | BaP Treatment:PCR Method | 0.13 | 1 | 0.717 | |
| Shannon | BaP Treatment | 2.45 | 1 | 0.117 | |
| | PCR Method | 0.07 | 1 | 0.796 | |
| | BaP Treatment:PCR Method | 0.01 | 1 | 0.909 | |
| Simpson | BaP Treatment | 1.98 | 1 | 0.159 | |
| | PCR Method | 0.01 | 1 | 0.907 | |
| | BaP Treatment:PCR Method | 0 | 1 | 0.973 | |
| Phylogenetic | BaP Treatment | 1.36 | 1 | 0.244 | |
| | PCR Method | 0.14 | 1 | 0.704 | |
| | BaP Treatment:PCR Method | 0.03 | 1 | 0.869 | |
| Observed | BaP Treatment | 0.87 | 1 | 0.35 | |
| | PCR Method | 0.13 | 1 | 0.716 | |
| | BaP Treatment:PCR Method | 0.25 | 1 | 0.616 | |

Supplemental Table 3. Alpha-diversity by extraction kit and dissection method GLM results (n = 222)

| metric | term | statistic | df | p.value | sig |
|---|---|---|---|---|---|
| Chao1 | BaP Treatment | 4.73 | 1 | 0.03 | * |
| | Extraction Kit | 173.76 | 2 | 0 | * |
| | Dissection Method | 22.06 | 1 | 0 | * |
| | BaP Treatment:Extraction Kit | 6.94 | 2 | 0.031 | * |
| | BaP Treatment:Dissection Method | 0.16 | 1 | 0.692 | |
| Shannon | BaP Treatment | 15.5 | 1 | 0 | * |
| | Extraction Kit | 78.42 | 2 | 0 | * |
| | Dissection Method | 16.09 | 1 | 0 | * |
| | BaP Treatment:Extraction Kit | 4.5 | 2 | 0.105 | |
| | BaP Treatment:Dissection Method | 2.3 | 1 | 0.129 | |
| Simpson | BaP Treatment | 11.28 | 1 | 0.001 | * |
| | Extraction Kit | 38.17 | 2 | 0 | * |
| | Dissection Method | 1.22 | 1 | 0.27 | |
| | BaP Treatment:Extraction Kit | 2.5 | 2 | 0.286 | |
| | BaP Treatment:Dissection Method | 2.06 | 1 | 0.151 | |
| Phylogenetic | BaP Treatment | 12.45 | 1 | 0 | * |
| | Extraction Kit | 233.9 | 2 | 0 | * |
| | Dissection Method | 13.36 | 1 | 0 | * |
| | BaP Treatment:Extraction Kit | 4.13 | 2 | 0.127 | |
| | BaP Treatment:Dissection Method | 0.03 | 1 | 0.867 | |
| Observed | BaP Treatment | 5.82 | 1 | 0.016 | * |
| | Extraction Kit | 177.55 | 2 | 0 | * |
| | Dissection Method | 26.18 | 1 | 0 | * |
| | BaP Treatment:Extraction Kit | 6.36 | 2 | 0.042 | * |
| | BaP Treatment:Dissection Method | 0.12 | 1 | 0.726 | |

Supplemental Table 4. PCR methods PERMANOVA results (n = 277)

| metric | term | df | SumOfSqs/Variance | statistic | p.value | sig |
|---|---|---|---|---|---|---|
| Bray-Curtis | BaP Treatment | 1 | 2.81 | 7.35 | 0.002 | * |
| | PCR Method | 1 | 0.08 | 0.21 | 0.002 | * |
| | BaP Treatment:PCR Method | 1 | 0.04 | 0.11 | 0.581 | |
| | Residual | 623 | 238.27 | | | |
| Canberra | BaP Treatment | 1 | 1.4 | 3.18 | 0.001 | * |
| | PCR Method | 1 | 0.4 | 0.9 | 0.001 | * |
| | BaP Treatment:PCR Method | 1 | 0.24 | 0.55 | 0.304 | |
| | Residual | 623 | 275.08 | | | |
| Sørensen | BaP Treatment | 1 | 1.98 | 6.09 | 0.001 | * |
| | PCR Method | 1 | 0.34 | 1.05 | 0.001 | * |
| | BaP Treatment:PCR Method | 1 | 0.16 | 0.48 | 0.054 | |
| | Residual | 623 | 202.77 | | | |
| Aitchison | BaP Treatment | 1 | 15.2 | 4.58 | 0.001 | * |
| | PCR Method | 1 | 2.78 | 0.84 | 0.001 | * |
| | BaP Treatment:PCR Method | 1 | 1.51 | 0.46 | 0.089 | |
| | Residual | 622 | 2,065.26 | | | |
| W Unifrac | BaP Treatment | 1 | 0.39 | 20.03 | 0.001 | * |
| | PCR Method | 1 | 0 | 0.19 | 0.001 | * |
| | BaP Treatment:PCR Method | 1 | 0 | 0.07 | 0.326 | |
| | Residual | 623 | 12 | | | |
| 0.5 Unifrac | BaP Treatment | 1 | 1.41 | 13.52 | 0.001 | * |
| | PCR Method | 1 | 0.04 | 0.39 | 0.001 | * |
| | BaP Treatment:PCR Method | 1 | 0.02 | 0.18 | 0.071 | |
| | Residual | 623 | 64.87 | | | |
| U Unifrac | BaP Treatment | 1 | 2.11 | 6.13 | 0.001 | * |
| | PCR Method | 1 | 0.34 | 0.98 | 0.001 | * |
| | BaP Treatment:PCR Method | 1 | 0.16 | 0.47 | 0.083 | |
| | Residual | 623 | 214.18 | | | |

Supplemental Table 5. Extraction kit and dissection methods PERMANOVA results (n = 222)

| metric | term | df | SumOfSqs/Variance | statistic | p.value | sig |
|---|---|---|---|---|---|---|
| Bray-Curtis | BaP Treatment | 1 | 0.83 | 2.54 | 0.005 | * |
| | Extraction Kit | 2 | 6.13 | 9.4 | 0.001 | * |
| | Dissection Method | 1 | 6.65 | 20.38 | 0.001 | * |
| | BaP Treatment:Extraction Kit | 2 | 0.76 | 1.16 | 0.239 | |
| | BaP Treatment:Dissection Method | 1 | 0.38 | 1.15 | 0.277 | |
| | Residual | 214 | 69.81 | | | |
| Canberra | BaP Treatment | 1 | 0.65 | 1.54 | 0.001 | * |
| | Extraction Kit | 2 | 4.13 | 4.94 | 0.001 | * |
| | Dissection Method | 1 | 2.68 | 6.4 | 0.001 | * |
| | BaP Treatment:Extraction Kit | 2 | 0.91 | 1.09 | 0.098 | |
| | BaP Treatment:Dissection Method | 1 | 0.49 | 1.18 | 0.053 | |
| | Residual | 214 | 89.58 | | | |
| Sørensen | BaP Treatment | 1 | 0.81 | 3.12 | 0.001 | * |
| | Extraction Kit | 2 | 8.48 | 16.28 | 0.001 | * |
| | Dissection Method | 1 | 4.09 | 15.7 | 0.001 | * |
| | BaP Treatment:Extraction Kit | 2 | 0.67 | 1.28 | 0.085 | |
| | BaP Treatment:Dissection Method | 1 | 0.34 | 1.3 | 0.113 | |
| | Residual | 214 | 55.74 | | | |
| Aitchison | BaP Treatment | 1 | 17.79 | 2.17 | 0.003 | * |
| | Extraction Kit | 2 | 212.71 | 12.95 | 0.001 | * |
| | Dissection Method | 1 | 129.6 | 15.78 | 0.001 | * |
| | BaP Treatment:Extraction Kit | 2 | 20.43 | 1.24 | 0.099 | |
| | BaP Treatment:Dissection Method | 1 | 11.22 | 1.37 | 0.078 | |
| | Residual | 213 | 1,749.26 | | | |
| W Unifrac | BaP Treatment | 1 | 0.12 | 7.39 | 0.001 | * |
| | Extraction Kit | 2 | 0.36 | 11.05 | 0.001 | * |
| | Dissection Method | 1 | 0.42 | 25.82 | 0.001 | * |
| | BaP Treatment:Extraction Kit | 2 | 0.04 | 1.37 | 0.151 | |
| | BaP Treatment:Dissection Method | 1 | 0.02 | 0.95 | 0.409 | |
| | Residual | 214 | 3.5 | | | |
| 0.5 Unifrac | BaP Treatment | 1 | 0.46 | 5.63 | 0.001 | * |
| | Extraction Kit | 2 | 2.96 | 18.23 | 0.001 | * |
| | Dissection Method | 1 | 2.23 | 27.45 | 0.001 | * |
| | BaP Treatment:Extraction Kit | 2 | 0.22 | 1.38 | 0.101 | |
| | BaP Treatment:Dissection Method | 1 | 0.13 | 1.57 | 0.09 | |
| | Residual | 214 | 17.37 | | | |
| U Unifrac | BaP Treatment | 1 | 0.84 | 2.77 | 0.001 | * |
| | Extraction Kit | 2 | 6.73 | 11.13 | 0.001 | * |
| | Dissection Method | 1 | 3.43 | 11.34 | 0.001 | * |
| | BaP Treatment:Extraction Kit | 2 | 0.71 | 1.17 | 0.113 | |
| | BaP Treatment:Dissection Method | 1 | 0.43 | 1.43 | 0.038 | * |
| | Residual | 214 | 64.68 | | | |

Supplemental Table 6. Results of two binomial GLM models (one for each dissection method), built in a serial, forward stepwise manner predicting BaP treatment from individual ASV abundances.

| data.set | term | statistic | df | p.value | sig |
|---|---|---|---|---|---|
| dissected | ASV00002 | 1,335.57 | 1 | 0 | * |
| | ASV00007 | 1,912.27 | 1 | 0 | * |
| | ASV00017 | 18.23 | 1 | 0 | * |
| | ASV00019 | 42.08 | 1 | 0 | * |
| | ASV00049 | 13.03 | 1 | 0 | * |
| | ASV00067 | 2.2 | 1 | 0.138 | |
| | ASV00075 | 11.98 | 1 | 0.001 | * |
| | ASV00078 | 21.8 | 1 | 0 | * |
| | ASV00087 | 1,263.48 | 1 | 0 | * |
| | ASV00095 | 4.31 | 1 | 0.038 | * |
| | ASV00098 | 22.48 | 1 | 0 | * |
| | ASV00103 | 27.09 | 1 | 0 | * |
| | ASV00109 | 5.52 | 1 | 0.019 | * |
| | ASV00131 | 1,191.39 | 1 | 0 | * |
| | ASV00138 | 7.45 | 1 | 0.006 | * |
| | ASV00140 | 10.56 | 1 | 0.001 | * |
| | ASV00219 | 11.08 | 1 | 0.001 | * |
| | ASV00225 | 7.96 | 1 | 0.005 | * |
| | ASV00266 | 12.65 | 1 | 0 | * |
| | ASV00298 | 8.21 | 1 | 0.004 | * |
| whole fish | ASV00004 | 32.04 | 1 | 0 | * |
| | ASV00014 | 25.03 | 1 | 0 | * |
| | ASV00024 | 34.19 | 1 | 0 | * |
| | ASV00029 | 20.91 | 1 | 0 | * |
| | ASV00032 | 57.77 | 1 | 0 | * |
| | ASV00038 | 32.7 | 1 | 0 | * |
| | ASV00046 | 37.83 | 1 | 0 | * |
| | ASV00092 | 14.92 | 1 | 0 | * |
| | ASV00093 | 11.56 | 1 | 0.001 | * |
| | ASV00099 | 24.96 | 1 | 0 | * |
| | ASV00110 | 30.36 | 1 | 0 | * |
| | ASV00117 | 34.72 | 1 | 0 | * |
| | ASV00182 | 31.43 | 1 | 0 | * |
| | ASV00239 | 22.82 | 1 | 0 | * |
| | ASV00240 | 20.57 | 1 | 0 | * |
| | ASV00324 | 15.75 | 1 | 0 | * |

Supplemental Table 7. Results of two binomial GLM models (one for each dissection method), built in a serial, forward stepwise manner predicting BaP treatment from individual ASV abundances.

| data.set | term | statistic | df | p.value | sig |
|---|---|---|---|---|---|
| dissected | ASV00011 | 39.2 | 1 | 0 | * |
| | ASV00017 | 33.78 | 1 | 0 | * |
| | ASV00018 | 42.81 | 1 | 0 | * |
| | ASV00028 | 58.23 | 1 | 0 | * |
| | ASV00032 | 70.57 | 1 | 0 | * |
| | ASV00033 | 72.52 | 1 | 0 | * |
| | ASV00041 | 34.75 | 1 | 0 | * |
| | ASV00045 | 38.52 | 1 | 0 | * |
| | ASV00049 | 28.05 | 1 | 0 | * |
| | ASV00061 | 43.83 | 1 | 0 | * |
| | ASV00071 | 22.74 | 1 | 0 | * |
| | ASV00073 | 50.89 | 1 | 0 | * |
| | ASV00075 | 47.71 | 1 | 0 | * |
| | ASV00087 | 34.96 | 1 | 0 | * |
| | ASV00095 | 23.97 | 1 | 0 | * |
| | ASV00106 | 29.8 | 1 | 0 | * |
| | ASV00182 | 73.23 | 1 | 0 | * |
| | ASV00220 | 1,946.36 | 1 | 0 | * |
| | ASV00222 | 36.8 | 1 | 0 | * |
| | ASV00244 | 27.22 | 1 | 0 | * |
| whole fish | ASV00003 | 0.75 | 1 | 0.386 | |
| | ASV00004 | 16.9 | 1 | 0 | * |
| | ASV00032 | 34.04 | 1 | 0 | * |
| | ASV00038 | 31.38 | 1 | 0 | * |
| | ASV00046 | 7.26 | 1 | 0.007 | * |
| | ASV00091 | 7.03 | 1 | 0.008 | * |
| | ASV00117 | 21.6 | 1 | 0 | * |
| | ASV00167 | 4.16 | 1 | 0.041 | * |
| | ASV00239 | 13.28 | 1 | 0 | * |
| | ASV00310 | 4.84 | 1 | 0.028 | * |
| | ASV00324 | 3.48 | 1 | 0.062 | |

Supplemental Table 8. Results of three binomial GLM models (one for each extraction kit), built in a serial, forward stepwise manner predicting BaP treatment from individual ASV abundances.

| data.set | term | statistic | df | p.value | sig |
|---|---|---|---|---|---|
| Blood & Tissue | ASV00011 | 0 | 1 | 0.999 | |
| | ASV00022 | 10.89 | 1 | 0.001 | * |
| | ASV00024 | 0 | 1 | 1 | |
| | ASV00032 | 19.06 | 1 | 0 | * |
| | ASV00038 | 25.86 | 1 | 0 | * |
| | ASV00041 | 21.02 | 1 | 0 | * |
| | ASV00046 | 0 | 1 | 0.999 | |
| | ASV00076 | 792.96 | 1 | 0 | * |
| | ASV00087 | 27.98 | 1 | 0 | * |
| | ASV00106 | 8.69 | 1 | 0.003 | * |
| | ASV00145 | 0 | 1 | 1 | |
| | ASV00208 | 21.03 | 1 | 0 | * |
| NucleoSpin | ASV00008 | 21.29 | 1 | 0 | * |
| | ASV00025 | 14.98 | 1 | 0 | * |
| | ASV00032 | 17.94 | 1 | 0 | * |
| | ASV00035 | 21.86 | 1 | 0 | * |
| | ASV00038 | 39.93 | 1 | 0 | * |
| | ASV00040 | 30.59 | 1 | 0 | * |
| | ASV00041 | 432.52 | 1 | 0 | * |
| | ASV00096 | 14.78 | 1 | 0 | * |
| | ASV00239 | 18.77 | 1 | 0 | * |
| | ASV00339 | 9.98 | 1 | 0.002 | * |
| | ASV00443 | 28.48 | 1 | 0 | * |
| PowerSoil | ASV00005 | 3.79 | 1 | 0.052 | |
| | ASV00007 | 15.62 | 1 | 0 | * |
| | ASV00029 | 5.13 | 1 | 0.023 | * |
| | ASV00039 | 5.62 | 1 | 0.018 | * |
| | ASV00043 | 17.61 | 1 | 0 | * |
| | ASV00045 | 18.34 | 1 | 0 | * |
| | ASV00062 | 1.57 | 1 | 0.211 | |
| | ASV00126 | 4.24 | 1 | 0.039 | * |
| | ASV00133 | 8.52 | 1 | 0.004 | * |
| | ASV00140 | 4.8 | 1 | 0.028 | * |
| | ASV00166 | 13.61 | 1 | 0 | * |
| | ASV00239 | 8.77 | 1 | 0.003 | * |

Supplemental Table 9. Results of three binomial GLM models (one for each extraction kit), built in a serial, forward stepwise manner predicting BaP treatment from individual ASV abundances.

| data.set | term | statistic | df | p.value | sig |
|---|---|---|---|---|---|
| Blood & Tissue | ASV00001 | 26.43 | 1 | 0 | * |
| | ASV00013 | 25.29 | 1 | 0 | * |
| | ASV00018 | 28.59 | 1 | 0 | * |
| | ASV00022 | 21.33 | 1 | 0 | * |
| | ASV00032 | 59.8 | 1 | 0 | * |
| | ASV00038 | 45.04 | 1 | 0 | * |
| | ASV00041 | 23.68 | 1 | 0 | * |
| | ASV00046 | 30.82 | 1 | 0 | * |
| | ASV00049 | 30.16 | 1 | 0 | * |
| | ASV00103 | 28.6 | 1 | 0 | * |
| | ASV00239 | 29.57 | 1 | 0 | * |
| | ASV00388 | 26.83 | 1 | 0 | * |
| NucleoSpin | ASV00015 | 20.18 | 1 | 0 | * |
| | ASV00024 | 0 | 1 | 0.999 | |
| | ASV00025 | 18.74 | 1 | 0 | * |
| | ASV00032 | 12.21 | 1 | 0 | * |
| | ASV00035 | 17.68 | 1 | 0 | * |
| | ASV00038 | 44.9 | 1 | 0 | * |
| | ASV00041 | 0 | 1 | 1 | |
| | ASV00046 | 22.33 | 1 | 0 | * |
| | ASV00049 | 0 | 1 | 0.984 | |
| | ASV00093 | 0 | 1 | 0.999 | |
| | ASV00133 | 18.63 | 1 | 0 | * |
| | ASV00443 | 29.36 | 1 | 0 | * |
| PowerSoil | ASV00021 | 6.24 | 1 | 0.013 | * |
| | ASV00038 | 0 | 1 | 1 | |
| | ASV00039 | 17.48 | 1 | 0 | * |
| | ASV00040 | 4.48 | 1 | 0.034 | * |
| | ASV00045 | 19.26 | 1 | 0 | * |
| | ASV00046 | 15.95 | 1 | 0 | * |
| | ASV00058 | 0.98 | 1 | 0.323 | |
| | ASV00060 | 20.36 | 1 | 0 | * |
| | ASV00084 | 27.18 | 1 | 0 | * |
| | ASV00099 | 1.16 | 1 | 0.282 | |
| | ASV00101 | 21.69 | 1 | 0 | * |
| | ASV00133 | 15.84 | 1 | 0 | * |
| | ASV00139 | 12.74 | 1 | 0 | * |
